# Enhancing sit-to-stand transitions and walking efficiency in older adults with a soft robotic suit

**DOI:** 10.1038/s41467-026-75528-1

**Published:** 2026-07-17

**Authors:** Xiaohui Zhang, Enrica Tricomi, Marios Stefanakis, Nathalie Gierden, Theresa Buchner, Luka Mišković, Jürgen M. Bauer, Christian Werner, Clemens Becker, Lorenzo Masia

**Affiliations:** 1 Department of Computer Engineering, School of Computation, Information and Technology, Technical University of Munich, Munich, Germany; 2 Geriatric Center, Medical Faculty Heidelberg, Heidelberg University, Heidelberg, Germany; 3 Network Aging Research, Heidelberg University, Heidelberg, Germany

**Keywords:** Biomedical engineering, Health care, Electrical and electronic engineering, Geriatrics

## Abstract

Age-related declines in muscle strength and neuromuscular control make sit-to-stand transitions and walking progressively more difficult, compromising mobility and independence. Although wearable assistive technologies have been proposed to alleviate these challenges, few have demonstrated clear benefits in facilitating sit-to-stand movements for older adults who retain a degree of independent mobility. Here, we introduce a soft hip exosuit designed to assist both sit-to-stand transitions and walking activities. In a feasibility study involving ten older adults, the exosuit increased 1-minute sit-to-stand repetitions by an average of 1.8 and reduced the metabolic cost of walking by 13.6% compared with the unassisted condition. These improvements were achieved while preserving natural kinematics, lower-limb stability, and maintaining a strong sense of agency. Our findings demonstrate that soft exosuits can enhance sit-to-stand and walking performance in older adults while preserving biomechanical naturalness and user autonomy, highlighting their potential for practical home-integrated mobility assistance.

## Abstract (web summary)

Declines in muscle strength make sit-to-stand transitions and walking difficult for older adults. Here, the authors demonstrate that a soft hip exosuit improves sit-to-stand performance and reduces the metabolic cost of walking while preserving natural movement and users sense of agency.

## Introduction

Mobility, particularly the ability to rise from a chair and to walk, is fundamental to independent living and quality of life. However, age-related losses in muscle strength and neuromuscular coordination make these everyday tasks increasingly demanding, limiting outdoor activity, accelerating functional decline, and elevating fall risk^1,2^. These challenges motivate assistive technologies that can support mobility while remaining compatible with natural movement in community-dwelling older adults.

Soft wearable assistive exosuits address this need by delivering compliant, body-conforming assistance without the bulk and kinematic constraints of rigid exoskeletons^3,4^. Their potential is particularly evident in assisting walking^5–9^. For instance, soft exosuits assisting the hip or ankle joint have been shown to reduce the metabolic cost of walking and modulate gait mechanics in healthy young and older adults, as well as in individuals with post-stroke hemiparesis^10–12^, highlighting the suitability of soft structures for cyclic assistance and further demonstrating the broad potential of soft wearable devices to augment human locomotion.

Alongside walking, sit-to-stand transitions are among the most demanding and frequently performed activities of daily living, particularly for older adults^13^, yet it involves non-cyclic, transient movements with rapid, phase-dependent changes in hip joint power requirements. These dynamics pose fundamental challenges for soft wearable exosuits, which need to deliver biomechanically meaningful assistance under limited structural rigidity and actuation bandwidth. To date, sit-to-stand assistance technologies have largely followed rigid, joint-actuated exoskeleton paradigms, exemplified by systems such as Hybrid Assistive Limb (HAL) and powered knee devices^14–17^. These devices were primarily developed for users with substantial motor impairments and have demonstrated improvements in sit-to-stand performance in clinical populations, such as stroke survivors. However, their mass, structural stiffness, and reliance on external support constrain their suitability for community-dwelling older adults, who typically retain basic mobility but may experience age-related declines in perceived physical capability and confidence, thereby requiring lightweight, compliant, and socially acceptable assistance for everyday use^3,14^.

Existing soft wearable systems for sit-to-stand assistance are predominantly passive, relying on elastic elements to store and release energy (e.g., X-tights^18^ and Rakunie RK2^19^). In these systems, assistive force magnitude and timing are coupled to joint kinematics and elastic deformation, limiting independent control and the ability to accommodate rapidly changing assistance demands during sit-to-stand transitions, thereby reducing wearer comfort and adaptability in daily mobility tasks. Active tendon-driven soft exosuits offer the potential to overcome these limitations by enabling precise modulation of assistive forces while supporting both sit-to-stand transfers and walking within a lightweight wearable system. Addressing these challenges may enable broader adoption of soft wearable assistive technologies and help mitigate age-related declines in functional mobility.

**Fig. 1:**
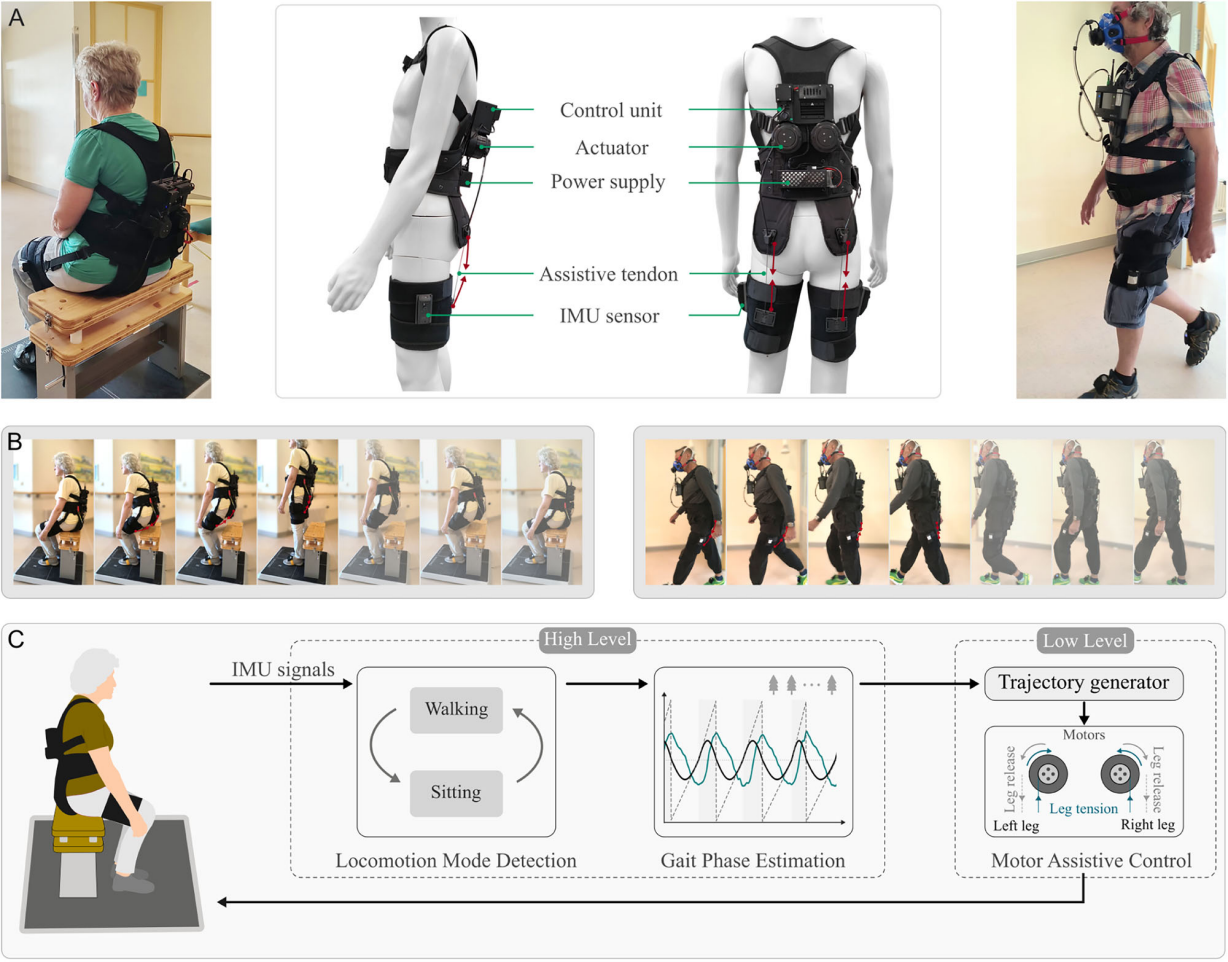
Soft wearable exosuit. **A** Exosuit consists of textile components (vest, waist belt, and thigh harnesses) and integrated mechatronic modules (control unit, actuators, power supply, assistive tendons, and IMU sensors), which assist with the lower-limb during sit-to-stand transition and walking tasks via artificial tendons. **B** Time-lapse sequences of older adult participants performing sit-to-stand transitions and overground walking tasks while wearing the exosuit. **C** Thigh-mounted inertial measurement unit (IMU) sensors capture kinematic signals, which are processed by a high-level module for locomotion mode detection and gait phase estimation to drive the lower-level actuators for delivering appropriate assistive forces to the user.

In this study, we present a soft tendon-driven hip exosuit designed to support sit-to-stand transitions and walking for older adults with reduced lower-limb functional performance (Fig. 1). By transmitting assistive forces through artificial tendons, the exosuit aims to provide biomechanically meaningful hip extension assistance during both sit-to-stand transitions and walking, while maintaining a soft, clothing-compatible form for everyday use. We hypothesize that the soft exosuit would improve sit-to-stand performance and reduce the metabolic cost of walking in older adults. We further hypothesize that the exosuit would improve movement efficiency while maintaining natural lower-limb biomechanics, stability-related performance, and wearers’ ability to actively control their movements.

The main contribution of this work is the demonstration of a lightweight, tendon-driven hip exosuit that extends soft wearable assistance from cyclic walking to sit-to-stand functional mobility in older adults with diminished lower-limb function. We conducted an evaluation of the exosuit and its task-oriented design and control framework in a cohort of older adults with a mean age of 77.7 ± 6.1 years. The evaluation supported our hypotheses and provided preliminary evidence of its functional, energetic, and agency-related impacts in the older adult population. Together, these results expand lower-limb soft wearable assistance beyond cyclic walking to dynamic, multifunctional tasks and provide a foundation for future translational studies.

## Results

### Participants

Ten participants were recruited (6 females and 4 males; age 69–85 years, mean 77.7 ± 6.1 years; height 169.3 ± 6.9 cm; weight 69.1 ± 9.2 kg, mean ± SD). Participants showed reduced lower-limb functional performance, indicated by the five-times sit-to-stand (FTSS) time of 13.6 ± 3.1 s^20^ and the Clinical Frailty Scale (CFS) score of 2.6 ± 0.8^21^.

### Sit-to-stand efficiency was found to be significantly improved in older adults

**Fig. 2:**
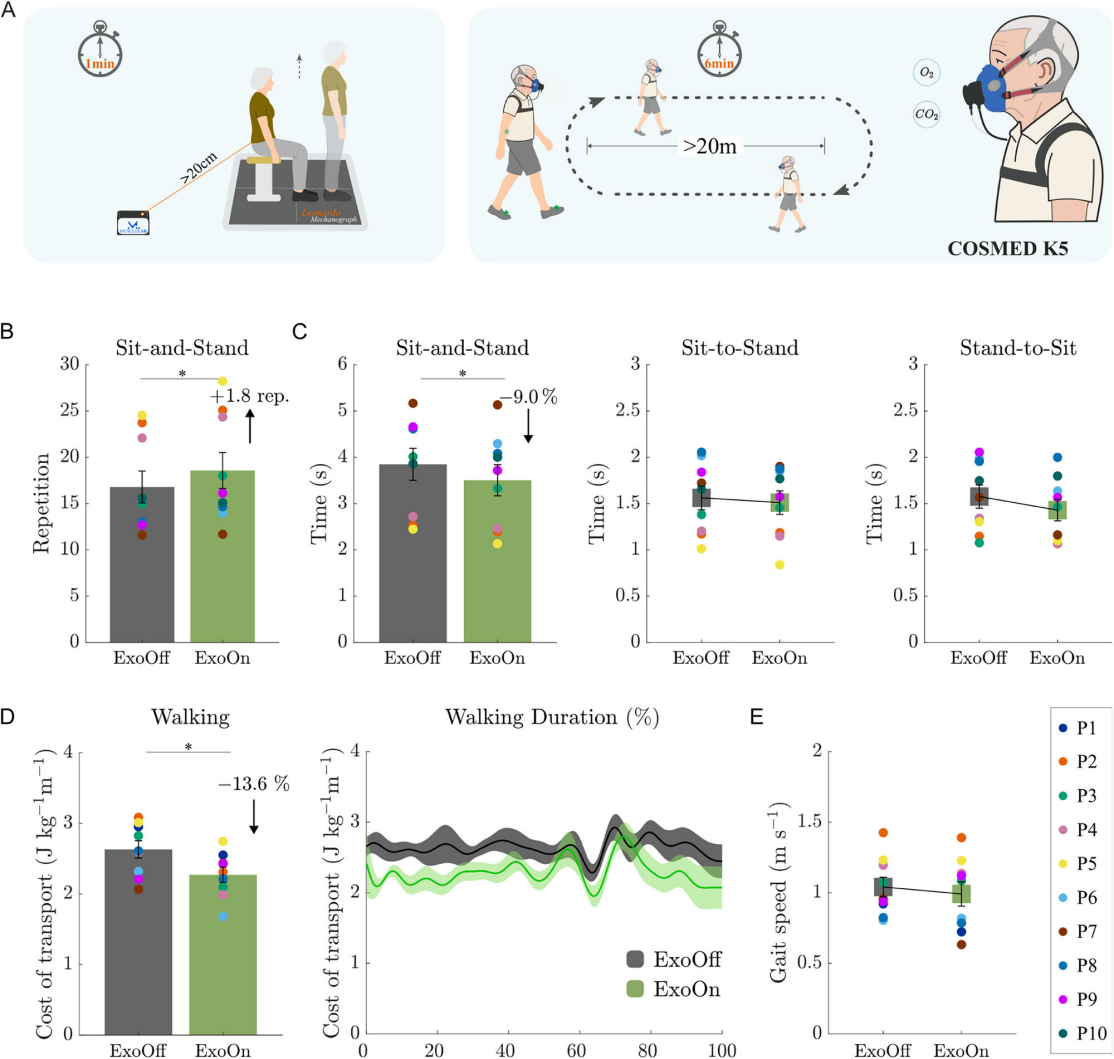
Experiment and performance evaluation of the exosuit in older adults (*n* = 9). **A** Experiment overview of sit-to-stand and walking. Sit-to-stand performance evaluation in 1-min sit-to-stand test: **B** the total repetitions and **C** time of each sit-and-stand cycle and transitions across participants. Walking performance evaluation in 6-min walking test: **D** the walking metabolic cost of transport and **E** average walking gait speed across participants under the *ExoOff* (unassisted) and *ExoOn* (assisted) conditions. The bar and curve plots are presented as mean ± s.e.m., with gray and green bars denoting the *ExoOff* and *ExoOn* conditions, respectively. Colored dots overlaid on the bars represent individual participants. * indicates statistically significant differences (*p* < 0.05, paired two-tailed *t*-tests).

Physical performance tests are widely used to characterize multiple dimensions of lower-limb function in older adults^22^. In this study, participants were instructed to perform the 1-min sit-to-stand test (1MSTS)^23^, in which they conducted as many sit-and-stand cycles as possible within 1 min while ensuring their own safety. Each sit-and-stand cycle comprised a sit-to-stand and a stand-to-sit transition phase. The sit-to-stand movement is a fundamental activity of daily living, and the 1MSTS is commonly used to assess functional exercise capacity and lower-limb muscle endurance, with its performance considered relevant to activities of daily living^24^. Preliminary results showed that assistance provided by the exosuit significantly enhanced sit-to-stand transition performance. Compared with the unassisted condition (*ExoOff*), the exosuit-assisted condition (*ExoOn*) resulted in an average increase of 1.8 ± 0.5 additional cycles in 1MSTS (16.8 ± 1.7 rep. in *ExoOff*, 18.6 ± 1.9 rep. in *ExoOn*; *n* = 9, *p* = 0.006) (Fig. 2B). Eight participants exhibited shorter sit-to-stand times when using the exosuit, with the largest individual improvement corresponding to a 20.2% reduction in cycle duration (4.66 s with *ExoOff* and 3.72 s with *ExoOn*). Exosuit assistance reduced the duration of a single sit-and-stand cycle by 9.0% (*n* = 9, *p* = 0.015) (Fig. 2C), corresponding to an average shortening of approximately 350 ms per cycle. Phase-specific analyses further revealed a 3.0% reduction in sit-to-stand time and a 7.5% reduction in stand-to-sit time, indicating that the exosuit facilitated both components of the transitional movement. Individual sit-to-stand performance of older participants is further provided in Supplementary Fig. S3. Participant 1 (P1) completed only 30 s of the sit-to-stand test due to physical limitations and was therefore excluded from this analysis.

### Metabolic cost of transport during walking was significantly reduced with exosuit assistance

To evaluate the energetic effects of the exosuit assistance during walking, older adults completed a 6-min walking test (6MWT), a widely used assessment of functional walking capacity^25,26^. Walking energetic efficiency was quantified using the metabolic cost of transport^27^, defined as the energy expended per unit body mass and distance traveled. Exosuit assistance significantly reduced the metabolic cost of transport in older adults, with a mean reduction of 13.6% across participants (from 2.63 ± 0.12 Jkg^−1^ m^−1^ in *ExoOff* to 2.27 ± 0.11 Jkg^−1^ m^−1^ in *ExoOn*; *n* = 9, *p* = 0.030) (Fig. 2D). In addition, a non-significant reduction in self-selected walking speed by approximately 5.0% (*n* = 9) was observed under the *ExoOn* condition compared with the *ExoOff* condition (Fig. 2E). Additional individual metabolic cost of walking transport data for older participants are provided in Supplementary Fig. S4. Participant 10 (P10) was excluded from the metabolic cost analysis due to missing *ExoOff* metabolic cost data.

### Lower-limb natural kinematics and stability were largely preserved

**Fig. 3:**
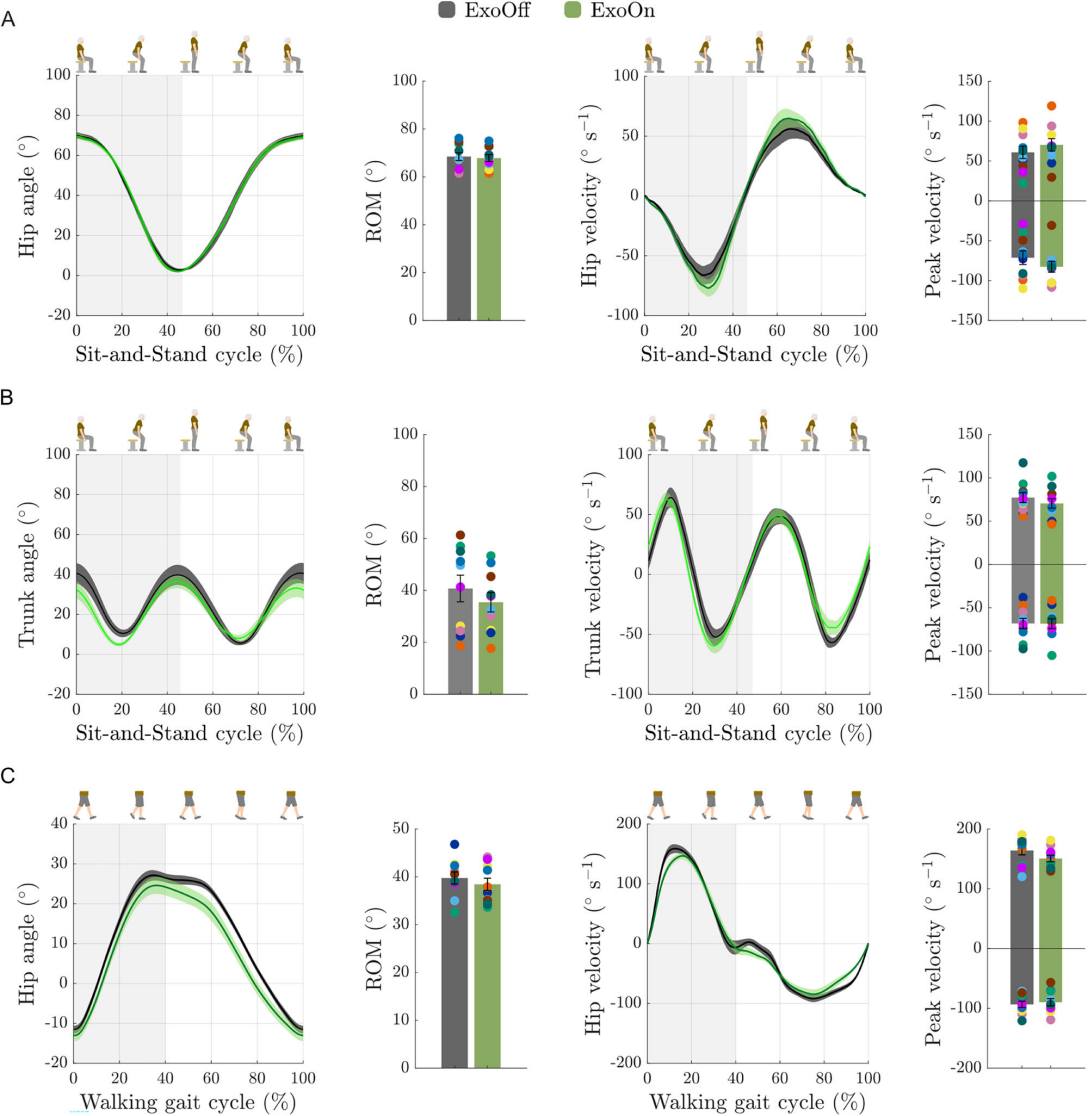
Kinematic evaluation during sit-to-stand and walking movements in older adults (*n* = 10). Angle and velocity profiles during sit-to-stand and walking tasks: **A** Hip and **B** trunk profiles during sit-and-stand cycles; **C** hip profile during walking. Range of motion (ROM) was calculated as the difference between the maximum and minimum angles within each segmented sit-and-stand or walking cycle across steps and participants. Velocity profiles were quantified by peak positive and peak negative velocity values. Bar and curve plots show the mean ± s.e.m. for the *ExoOff* (gray) and *ExoOn* (green) conditions, and overlaid dots represent data from individual participants.

Lower-limb kinematics and stability-related measures during sit-to-stand and walking were analyzed and compared between assisted and unassisted conditions to assess potential alterations in natural movement associated with exosuit assistance. Across both the sit-to-stand and walking tasks, hip joint and trunk kinematics were largely preserved with exosuit assistance. Hip and trunk angle trajectories, ranges of motion, and peak movement velocities showed only small observable changes and did not differ significantly between the *ExoOff* and *ExoOn* conditions (Fig. 3). During sit-to-stand task (Fig. 3A, B), hip angle range of motion decreased from 68.47 ± 1.66^∘^ in the *ExoOff* condition to 67.86 ± 1.45^∘^ in the *ExoOn* condition, and hip peak velocity increased from 60.90 ± 7.71^∘^ s^−1^ (*ExoOff*) to 70.11 ± 7.90^∘^ s^−1^ (*ExoOn*) for the maximum peak and from −71.25 ± 8.54^∘^ s^−1^ to −82.06 ± 7.29 ^∘^s^−1^ for the minimum peak; Trunk range of motion decreased from 40.75 ± 5.14^∘^ (*ExoOff*) to 35.45 ± 3.75^∘^ (*ExoOn*), while trunk peak velocity changed from 77.31 ± 5.79^∘^ s^−1^ to 70.57 ± 5.43^∘^ s^−1^ for the maximum peak and from −68.13 ± 5.90^∘^ s^−1^ to −63.38 ± 5.66^∘^ s^−1^ for the minimum peak. During walking (Fig. 3C), hip angle range of motion changed from 39.75 ± 1.27^∘^ to 38.35 ± 1.30^∘^, whereas hip peak velocity changed from 164.36 ± 7.52^∘^ s^−1^ to 150.28 ± 5.55^∘^ s^−1^ for the positive peak and from −93.36 ± 5.28^∘^ s^−1^ to −89.37 ± 6.10^∘^ s^−1^ for the negative peak, in the *ExoOff* and *ExoOn* conditions, respectively. The preserved hip and trunk kinematics under exosuit assistance also provided complementary information relevant to the interpretation of movement stability and postural-control-related behavior.

**Fig. 4:**
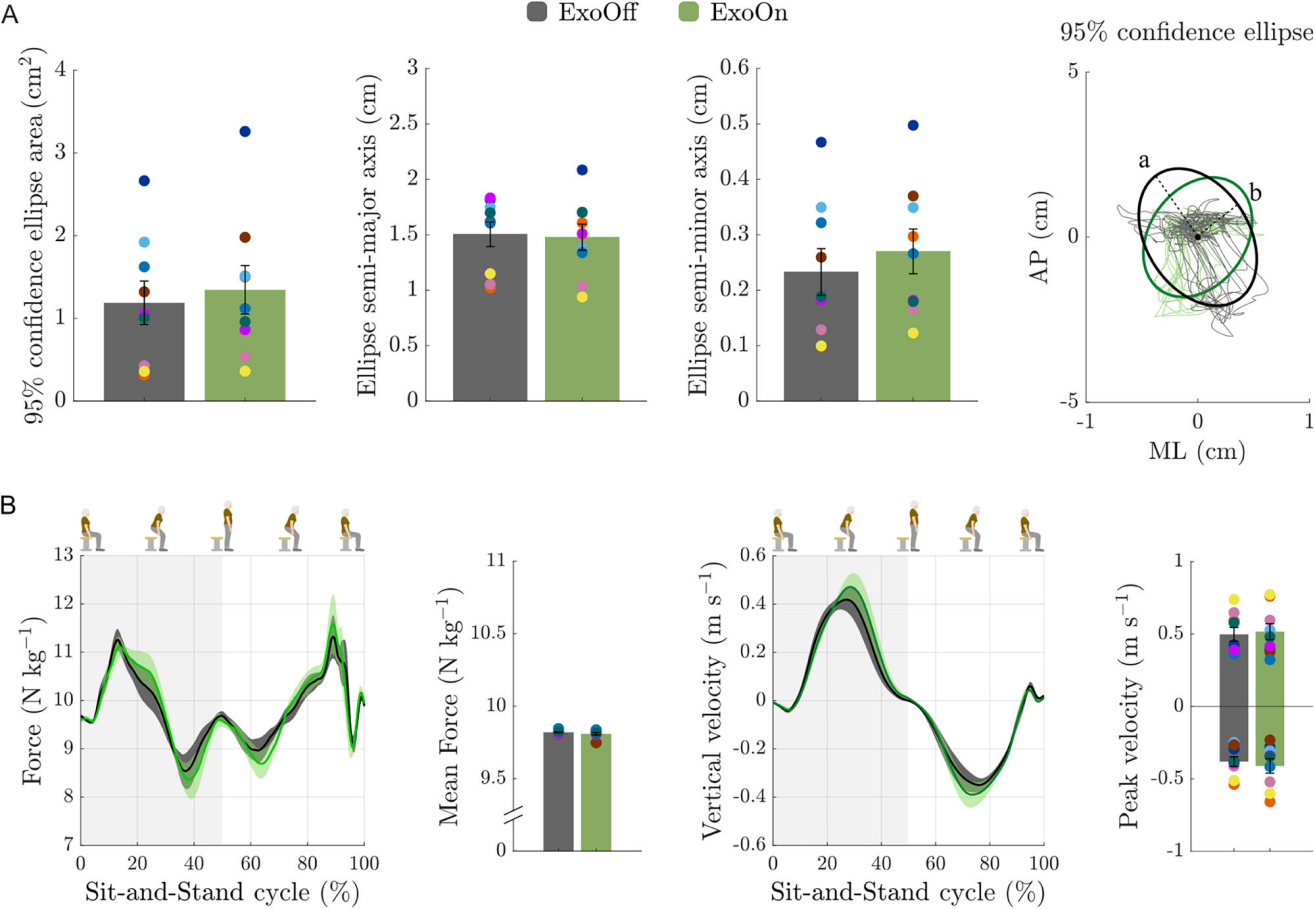
Biomechanical analysis and stability assessment of sit-to-stand movement in older adults through force plate metrics (*n* = 9). **A** 95% confidence ellipse fitted to center of force (COF) data points across participants during sit-to-stand test. The lengths of the semi-major axis (a) and semi-minor axis (b) also served as metrics for quantifying postural sway of the plantar pressure in the anteroposterior (AP) and mediolateral (ML) directions, respectively. Data from a representative participant (P1) are shown for illustration, with AP and ML coordinates zero-centered. **B** Trajectory and mean vertical ground reaction force and velocity across older participants. Bar and curve plots show mean ± s.e.m. for *ExoOff* (gray) and *ExoOn* (green) conditions; dots indicate individual participants.

To further characterize stability-related behavior during the sit-to-stand task, the 95% confidence ellipse of the center-of-force (COF) trajectory recorded from the ground reaction force platform was analyzed. The semi-major and semi-minor axes of the ellipse represented stability in the anteroposterior (AP) and mediolateral (ML) directions, respectively. As shown in Fig. 4A, the COF ellipse area was 1.18 ± 0.26 cm^2^ under the *ExoOff* condition and 1.34 ± 0.29 cm^2^ under the *ExoOn* condition, with no significant difference observed between the two conditions (*n* = 9). Participant 3 (P3) was excluded from the stability analysis due to missing corresponding ground reaction force data. Similarly, the semi-major axis length in the AP direction (1.51 ± 0.11 cm vs. 1.47 ± 0.12 cm) and the minor axis length in the ML direction (0.23 ± 0.04 cm vs. 0.27 ± 0.04 cm) did not differ significantly between *ExoOff* and *ExoOn* conditions (*n* = 9). Additionally, the total ground reaction force and center of mass velocity profiles (Fig. 4B) were highly similar across participants between *ExoOff* and *ExoOn* conditions. Mean force was 9.82 ± 0.004 Nkg^−1^ in *ExoOff* and 9.81 ± 0.009 Nkg^−1^ in *ExoOn*. Peak center of mass velocity showed similarly small differences, with maximum peaks of 0.50 ± 0.05 ms^−1^ and 0.52 ± 0.05 ms^−1^ and minimum peaks of − 0.38 ± 0.03 ms^−1^ and − 0.41 ± 0.05 ms^−1^ for *ExoOff* and *ExoOn*, respectively. Neither effect reached statistical significance. These results suggest that the exosuit improves sit-to-stand transfer efficiency without adversely affecting the measured movement and stability-related outcomes in older participants. Furthermore, movement smoothness before and after exosuit assistance was quantified using the spectral arc length (SPARC), with the results presented in the Supplementary Fig. S5.

### Sense of agency was preserved in older adults

**Fig. 5:**
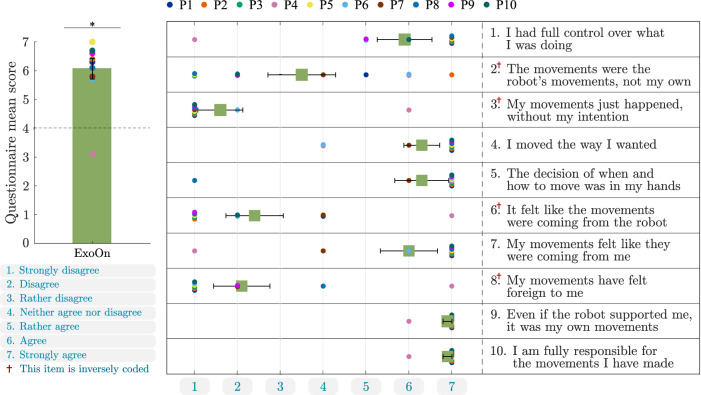
Sense of agency questionnaire in older adults (*n* = 10). Mean score (±s.e.m.; green bar) for each questionnaire item across all participants, along with the distribution of participant responses to each item (1–10) on the 7-point (ranging from -Strongly disagree- to -Strongly agree-) sense of agency questionnaire. The y-axis represents each question item, the x-axis represents the Likert response option, while colored circular markers denote individual participants' responses. Items 2, 3, 6, and 8 are inversely coded, whereas items 1, 4, 5, 7, 9, and 10 follow the standard scoring direction. * indicates a statistically significant difference from the scale midpoint of 4 (*p* < 0.05, two-tailed one-sample *t*-test).

Older adults reported a high perceived sense of agency during exosuit-assisted sit-to-stand transitions and walking movements, as assessed by a 10-item questionnaire, with a mean score of 6.07 ± 0.38, which was significantly higher than the neutral midpoint of 4 (*n* = 10, *p* < 0.001; Fig. 5). The scale showed good internal consistency, as indicated by Cronbach’s alpha (*α* = 0.86, 95% CI [0.68, 0.96]). Fig. 5 also shows the distribution (mean ± s.e.m.) of responses across individual items of the sense of agency questionnaire. For positively framed items (1, 4, 5, 7, 9, and 10), responses clustered toward the upper end of the 7-point scale, with the majority of participants selecting -Agree- or -Strongly agree-. In contrast, negatively framed items (2, 3, 6, and 8) showed response distributions weighted toward lower scores prior to reverse coding, indicating that participants generally rejected statements implying reduced control and reported a largely preserved sense of agency during *ExoOn* conditions. The percentage distribution of participants’ ratings on the sense of agency questionnaire is provided in Supplementary Fig. S6. The experimental details and results are further illustrated in Supplementary Movie 1.

## Discussion

As population ageing accelerates (by 2030, one in six people worldwide will be aged 60 years or older^28,29^), maintaining mobility and independent living in older adults is an increasingly important public health priority. Age-related declines in strength and coordination can make it difficult to rise from a chair and to sustain efficient walking, contributing to reduced activity and greater fear of falling. These challenges motivate wearable assistive technologies suitable for everyday use, and soft, textile-based exosuits are promising because they are lightweight, compliant, and can deliver assistance while preserving natural movement. For sit-to-stand assistance, most soft exosuits currently rely primarily on elastic elements^18,19,30^ that store energy during sitting and release it during rising. While this strategy reduces mass, the resulting restoring forces can increase device-body interface pressure during sitting, potentially constraining comfort and adaptability to individual movement strategies. In our previous study^31^, we assessed the effectiveness of an LM-Ease exosuit for sit-to-stand assistance in healthy participants. The present work instead advances soft exosuit research by evaluating whether a tendon-driven design can provide functional and energetic benefits for older adults with reduced lower-limb function during both sit-to-stand transitions and walking, while preserving natural movement patterns and a strong sense of agency. These insights inform the development of soft exosuits that can support older adults’ functional activities of daily living in home and community settings.

To this end, we propose and evaluate a soft hip exosuit designed to enhance sit-to-stand and walking performance in community-dwelling older adults. With assistance, older adults completed 1.8 ± 0.5 additional sit-and-stand cycles within 1 min, with a maximum increase of 4 cycles, and showed a 13.6% reduction in metabolic cost of transport during walking, with nearly half of participants showing reductions greater than 20%. Importantly, these functional and energetic gains were achieved while largely preserving natural lower-limb kinematics and the measured stability-related outcomes, indicating that assistance did not come at the cost of substantial alterations in movement behavior. The high perceived sense of agency further suggests that participants experienced the exosuit as support for their own movements rather than as a device that takes over control. Such outcomes are critical for older adults with reduced lower-limb function to maintain independence and social participation, and align directly with the goals of ageing in place.

The improvement in the number of sit-and-stand transition cycles (1.8 ± 0.5 additional cycles) completed within 1 min, together with the reduction of 9.0% in the duration of individual cycles, suggests that the soft exosuit can mitigate limitations associated with lower-limb weakness in this population. Beyond the immediate performance gains with the exosuit, easier and faster sit-to-stand transitions may also influence behavioral patterns over longer time scales. When older adults perceive rising from a chair as effortful or unsafe, they often limit how frequently they stand up, which in turn leads to prolonged sedentary time and further deconditioning. By lowering the perceived difficulty of the task, the exosuit has the potential to disrupt this negative cycle and encourage more frequent sit-to-stand transitions. Despite the clear group-level improvements, individual responses to the exosuit varied considerably (Supplementary Fig. S3). The number of completed sit-and-stand transition cycles ranged from no change to an increase of 4 cycles across participants (up to a 33.3% improvement relative to *ExoOff* condition). Correspondingly, relative changes in cycle duration ranged from a 3.7% increase to a 21.9% decrease, with one participant showing a slight slowing. These results highlight heterogeneous individual responses to exosuit hip assistance.

The metabolic cost of transport is commonly adopted as a primary outcome measure for assessing the effectiveness of wearable assistive devices^6,8,12,32^. Lower metabolic cost values indicate that an assistive device allows users to walk with greater energetic efficiency and therefore provides a direct proxy for improved gait economy and functional walking capacity. The observed reductions in metabolic cost of transport with the exosuit indicate improved walking efficiency in older adults, with an average 13.6% decrease. Given their typically reduced aerobic reserve, lowering the energetic cost of walking may delay the onset of fatigue and thereby support longer walking durations or greater distances in daily life^33,34^. Individual walking metabolic cost of transport profiles for older participants are shown in Supplementary Fig. S4. The metabolic cost of transport decreased in 7 of 9 participants (−8.8–−27.5%), with 4 participants showing reductions >20%, whereas 2 participants exhibited increases (9.9% and 18.0%). Such inter-individual variability across tasks may reflect differences in baseline functional capacity and balance confidence, preferred movement strategies, and the degree of biomechanical alignment between a generic assistance profile and individual biomechanics. Variations in donning/fit and short-term familiarization or adaptation to the exosuit may further contribute to response heterogeneity.

Faster sit-to-stand transitions may increase demands on postural control. In the present study, exosuit assistance improved sit-to-stand performance and walking efficiency while largely preserving natural lower-limb kinematics and postural stability. However, individual responses were heterogeneous. For instance, participant 2 (P2) completed three additional cycles within 1 min and increased sit-to-stand transition speed by 16.1%, accompanied by a trend toward reduced stability (ellipse area: from 0.31 cm^2^ (*ExoOff*) to 1.50 cm^2^ (*ExoOn*)), whereas P8 completed one additional cycle and increased speed by 11.0% while showing an opposite trend (ellipse area: from 1.62 cm^2^ to 1.12 cm^2^). This heterogeneity suggests that exosuit assistance may interact with individual movement strategies in different ways, even when group-level effects remain unchanged. Lower-limb and trunk kinematic analysis further supports the result that exosuit assistance did not substantially alter the fundamental movement pattern of the sit-to-stand movement or markedly compromise stability. However, it should be noted that the 95% confidence ellipse area of the center of force (COF) trajectory, used here as a stability-related measure during repeated sit-and-stand cycles, primarily reflects foot-ground interaction characteristics. A more comprehensive assessment of whole-body dynamic stability would require additional biomechanical measures, including center-of-mass displacement and velocity and continuous changes in the relationship between the center of mass and the base of support during the sit-to-stand movement. Consistent with these findings, exosuit assistance significantly improved walking smoothness, as quantified by SPARC (Supplementary Fig. S5), whereas smoothness during sit-to-stand transitions showed only a non-significant decreasing trend. Together with the largely preserved lower-limb biomechanics, these results suggest that the exosuit enhanced functional performance without substantially altering the underlying movement patterns. Substantial deviations in joint kinematics or ground reaction forces would typically indicate compensatory movement strategies that could increase joint loading or impose greater demands on balance control. However, the high similarity in hip and trunk trajectories and overall movement profiles between the *ExoOff* and *ExoOn* conditions suggests that the exosuit functioned more as an augmentation of existing movement patterns than as a driver of new ones. This biomechanical transparency is particularly desirable in older adults, whose capacity for motor adaptation may already be reduced and for whom substantial alterations in gait or sit-to-stand transition strategy could increase the risk of falls.

In addition to the improvements in sit-to-stand transition efficiency and walking energetics achieved with the exosuit, questionnaire responses also consistently indicated a strong perceived sense of agency during exosuit-assisted movement. For older adults, this subjective experience is especially important, as concerns about losing control and personal agency have been repeatedly identified as key factors influencing acceptance of wearable assistive robots^12,35^. The high agency ratings observed with the exosuit suggest that its tendon-driven hip assistance is sufficiently transparent to remain aligned with users’ natural motor intentions. By applying force in a manner that complements rather than dictates movement, the exosuit preserves the coherence between motor commands and sensory feedback, which is essential for maintaining a stable sense of volitional control.

A limitation of this study is that the primary experimental design compared only two wearing conditions: *ExoOff* (device worn with assistance disabled) and *ExoOn* (device worn with assistance enabled), without including a no-exosuit condition in the full participant cohort. To further contextualize the effect of active assistance relative to natural, unassisted movement, we also conducted a supplementary experimental session with a subgroup of 7 of the 10 originally enrolled participants, in which the same tasks were completed without wearing the exosuit (*NoExo*). The supplementary findings with *NoExo* and *ExoOn* were broadly consistent with the comparison between the *ExoOff* and *ExoOn* conditions. Specifically, compared with *NoExo*, the *ExoOn* condition significantly increased 1-min sit-to-stand performance by an average of 1.5 repetitions (*p* = 0.017) and significantly reduced the metabolic cost of transport during the 6-min walking test by 18.0% (*p* < 0.001) across all the participants, which is comparable to the corresponding improvements of 1.8 repetitions and 13.6% observed under *ExoOff* and *ExoOn* conditions. Given that the exosuit is a soft, lightweight textile-based system (2.86 kg), with its mass concentrated near the user’s center of mass rather than distally on the lower limbs, passive interference from device wearing is expected to be limited. This interpretation is consistent with our previous observations and related work^8,12^. Together with the comparable outcomes observed in the *NoExo* analysis, this supports the use of the *ExoOff* condition as a plausible reference baseline for assessing the effect of active assistance. Notably, similar experimental paradigms have been widely adopted in related studies^3,9,36^. The detailed results related to *NoExo* are presented in Supplementary Information.

Another point to note is that the sit-to-stand transition begins from a highly flexed posture, where lower-limb leverage is less favorable and effective mechanical advantage may be reduced, particularly around seat-off. Although exosuit assistance produced measurable functional benefits during sit-to-stand transitions, assisting this task with a soft tendon-driven device is inherently challenging from a biomechanical perspective. However, the exosuit was not designed to fully generate the movement or replace the user’s voluntary effort. Instead, assistance was delivered after recognition of the user’s movement intention and was intended to provide partial hip-extension support during the transition. Thus, movement initiation and the early preparatory phase remained primarily user-driven, while the exosuit contributed assistance during the subsequent extension-dominant phase. The observed improvement in 1-min sit-to-stand repetitions should therefore be interpreted as a functional benefit of cooperative partial assistance, rather than full mechanical replacement of lower-limb effort. Future studies that combine inverse dynamics with biomechanical modeling could help further clarify how joint mechanics, leverage, and effective mechanical advantage change across different movement phases, thereby strengthening the mechanistic interpretation of the observed improvements and informing the design of wearable assistive systems. Moreover, it should be noted that sit-to-stand and walking movements involve different mechanical and neuromuscular demands. Although both activities benefited from exosuit assistance under a task-oriented control framework, task-specific demands may lead to distinct interactions between the exosuit and the musculoskeletal system. Future studies incorporating electromyography or musculoskeletal modeling would help clarify how exosuit assistance influences musculotendon function differently during sit-to-stand and walking tasks. A further limitation is that we evaluated the exosuit’s assistance in older adults only during short laboratory tasks. Consequently, it remains unclear whether these benefits persist in daily life during continuous and multi-task scenarios (e.g., prolonged walking interleaved with sit-to-stand transitions), or how long-term use shapes user adaptation, tolerance, and acceptance. Future work will conduct longitudinal studies, quantifying changes in kinematics and metabolic cost during extended everyday activities in real-world or near-real-world settings, alongside systematic assessments of usability, comfort, and user acceptance. Importantly, moving toward such real-world deployment also motivates a closer examination of how assistance reshapes gait mechanics across individuals. Some older participants with an asymmetric gait reported that the exosuit helped the weaker leg adopt a more symmetric walking pattern. This positive feedback motivates future work to examine whether exosuit assistance can systematically enhance gait symmetry and load redistribution in individuals with pronounced asymmetry or unilateral weakness, and whether such changes confer measurable benefits for joint loading, perceived stability, and functional rehabilitation outcomes. Future studies should also characterize inter-individual variability and incorporate online learning or adaptive optimization to personalize assistance parameters to each user’s needs and usage patterns, providing a foundation for translating short-term experimental gains into stable, sustained functional improvements and ultimately enabling reliable assistance in older adults’ daily lives.

Looking ahead, soft exosuits, which are lightweight, body-conforming, and visually unobtrusive while improving both sit-to-stand efficiency and walking economy, may evolve into practical everyday supports for older adults. By reducing the physical demands of routine activities such as rising from a chair, walking to nearby shops, and participating in social events, these systems could help improve or maintain mobility in individuals who are still living independently and, over the longer term, contribute to preventing or slowing mobility loss in later life.

## Methods

### Exosuit hardware

The exosuit uses artificial tendons to provide hip extension assistance in response to the user’s movement patterns, enabling assistance during both sit-to-stand transitions and walking. Fig. 1A, B illustrate older adult participants wearing the exosuit while performing sit-to-stand and walking tasks, respectively. The central schematic of Fig. 1A depicts the main components of the system, including the control unit, actuator, power supply, assistive tendons, and IMU sensors integrated into a lightweight textile structure. The detailed schematics and locations of the hardware components are illustrated in Supplementary Fig. S1.

The inertial measurement unit (IMU) sensor modules mounted on the left and right lateral sides of the thighs transmit the recorded user kinematic signals to the control board via Bluetooth Low Energy (BLE) protocol. The algorithms running on the control board recognize the user’s locomotion mode and gait phase according to the current user kinematics information, and then generate the corresponding actuator reference signals to guide the motor operation (AK60-6, CubeMars actuator, China). The motor actuation drives a 3D-printed pulley mounted above the motor to rotate, causing the artificial assistive tendons (black woven Kevlar, USA) wrapped around the pulley to tighten and relax alternately. The resulting forces are transmitted through the Bowden sheaths (Shimano SLR, 4 mm diameter, Japan) to the 3D-printed anchor points fixed at the hip (proximal anchor points) and the thigh (distal anchor points). The main hardware components of each IMU module are a Feather board (BLE, Feather nRF52 Bluefruit, Adafruit, USA), a BNO055 board (Bosch, BNO055, Germany), and a battery (Lithium-Ion polymer battery, China). The Feather board serves to generate and send BLE signals, the BNO055 board is utilized for measuring kinematic signals, and the battery powers the entire IMU module.

The control unit module mounted on the back primarily consists of a Nvidia Jetson board (Nvidia Jetson Nano, USA), an Arduino board (Arduino MKR 1010 WiFi, Italy), a Can-bus extension board (Can-bus Shield V2.0, Seeed Studio, China), and a Feather board (BLE, Feather nRF52 Bluefruit, Adafruit, USA). The Nvidia Jetson board is capable of handling computationally demanding control algorithms and machine-learning-based algorithms while simultaneously collecting experimental data. The Arduino and Can-bus boards manage tasks such as reading sensor signals, executing less demanding control algorithms, and directing motor operations. The Feather board is utilized to receive BLE signals from IMU modules.

All electrical components in the control unit module are powered by a 14.8 V battery (Tattu, 14.8 V, 3700 mAh, 45 C, USA) located beneath it, either directly or after passing through a buck power converter board (24 V/12 V to 5 V 5 A DC-DC, China).

### Controller

The exosuit operates under a hierarchical control framework designed to provide adaptive assistance based on the lower-limb kinematics of the user, as shown in Fig. 1C. The system architecture follows a closed-loop control strategy, progressing from real-time IMU sensor signals acquisition to control decision-making and finally to actuator assistance. Kinematic data are continuously collected via two IMUs mounted on the thigh braces. At the high-level control layer, the kinematic data are processed to identify locomotion modes (e.g., sitting or walking) and to estimate the gait phase. Based on this estimation, the low-level control layer generates reference trajectories for the actuators tailored to the current locomotion phase and executes corresponding actuator commands to provide targeted assistance to the user. This control architecture enables the exosuit to facilitate natural and timely movement during daily activities. Detailed algorithmic descriptions of the control framework are provided further below and in Supplementary Fig. S2.

The high-level layer of the controller includes a locomotion mode recognition module and a gait phase estimation module. It utilizes IMU sensor data to identify the user’s movement state and guide the timing of assistance. The locomotion mode recognition module in the exosuit is implemented as a finite state machine (FSM) to enable real-time classification of user activities, such as sitting and walking. It uses two IMUs mounted on the user’s thighs to acquire key kinematic parameters, including the angles and movement velocities of the left and right thighs. The state transition logic is implemented using the *Chart* block in the Stateflow toolbox of MATLAB R2021b environment, enabling seamless integration with the overall control architecture. The decision logic is based on two computed features: the sum of hip angles (AngSum) and the sum of angular velocities (VelSum). These features were selected to provide a compact yet informative representation of the user’s lower-limb posture and dynamic state. Summing signals from both thighs helps mitigate the effects of transient asymmetrical limb movements, thereby improving the reliability of locomotion mode recognition. The FSM comprises two states: State 1, which includes the Reset and Walking sub-states, and State 2, corresponding to the Sitting state. The system initializes in the Reset sub-state of State 1, representing an upright posture at the onset of locomotion, and remains in the Walking sub-state of State 1 as long as the composite joint angle satisfies AngSum≤0.8 rad. The system switches from Walking to Sitting when AngSum > 0.8 rad and the composite angular velocity VelSum ≥ 0 rad s^−1^, indicating a continued increase in joint flexion characteristic of the descent into a seated posture. For sit-to-stand movement, this composite angle threshold corresponds to approximately 25% of the sit-to-stand transition progression, or about 23^∘^ for each thigh, and partial hip-extension assistance is provided during the remaining phase of the transition. The transition back to Walking is triggered when AngSum ≤ 2 rad and VelSum ≤ 0 rad s^−1^, corresponding to the restoration of an upright posture after completion of the sit-to-stand transition. All threshold values were empirically determined by analyzing the correlation and variability of kinematic features across different locomotion modes, sensor noise, and hardware settings, and were guided by prior domain knowledge to optimize control performance and generalization. The gait phase estimation module of the exosuit employs a random forest regression algorithm^37,38^, which extracts bilateral lower-limb kinematic (hip angle and velocity as input) features from IMUs mounted on the user’s thighs to enable real-time, continuous prediction of the gait phase (output). The gait phase estimation model was trained^31^ using data collected from eight healthy adults (six males, two females; average age 25.9 ± 1.8 years) in a data collection-oriented experimental session. Detailed training data collection and model training process are provided in the Controller Implementation section in the Supplementary Information.

The low-level controller generates customized reference trajectories for the actuator based on the outputs of the high-level locomotion mode and gait phase estimation model. A proportional controller with a first-order low-pass characteristic then tracks these trajectories by converting the error between the desired and actual motor positions into a motor angular velocity command, thereby driving the actuators to deliver assistive forces to the user.

### Experiment protocol and data processing

The experiment was conducted as a controlled trial aimed at evaluating the effectiveness of the exosuit device in assisting older adults with sit-to-stand and walking activities.

Ten participants were enrolled in the study, with a mean age of 77.8 ± 6.1 years. Detailed participant information is provided in Table 1. Nine participants exhibited a five-times sit-to-stand (FTSS) time of ≥10 s^20^. Frailty status was also assessed by using the Clinical Frailty Scale (CFS)^21^, providing complementary information^22^: five participants classified themselves as level 3 (-well, with treated comorbid disease-), three as level 2 (-well-), one as level 4 (-apparently vulnerable-), and one as level 1 (-very fit-).

**Table 1:** Older participants information

Participant	Sex (F/M)	Age (year)	Weight (kg)	Height (cm)	FTSS (≥ 10 s)	CFS
P1	M	84	73	178	Y	3
P2	F	82	66	164	Y	3
P3	F	69	75	176	Y	3
P4	M	85	61	172	Y	3
P5	M	76	70	170	N	1
P6	F	85	65	165	Y	4
P7	F	76	84	172	Y	2
P8	F	78	53	170	Y	3
P9	F	71	64	154	Y	2
P10	M	71	80	172	Y	2

#### Ethics

Prior to the experiment, each participant provided written informed consent, including consent for the publication of identifiable images. All procedures were conducted in accordance with the principles of the Declaration of Helsinki. The Ethical Committee of the Medical Faculty Heidelberg approved this research (resolution S-313/2020).

#### Experiment design protocol

Participants were asked to complete two functional mobility tests while wearing the exosuit under two experimental conditions: *ExoOff* (in which no assistive force was provided) and *ExoOn* (in which assistive forces were generated by the exosuit device). Prior to the start of the experiment, an instructor introduced the operational principles of the exosuit system and demonstrated the required tasks. Participants subsequently donned the exosuit and practiced the sit-to-stand and walking tasks until they were fully familiarized with the procedure. The familiarization period included at least 5 sit-to-stand repetitions and 20 m of walking. A trained examiner closely supervised the entire testing procedure to ensure participant safety and compliance with instructions.

1-Min Sit-To-Stand test (1MSTS; Fig. 2A): Participants were required to perform as many sit-and-stand cycles as possible within 1 min period while maintaining balance and a natural movement rhythm. Prior to testing, they donned the exosuit device and were seated on a 46 cm armless chair integrated with a ground reaction force platform (Leonardo Mechanography, Novotec Medical, Germany). Their feet were positioned flat on the designated left and right force-sensing areas, naturally spaced at shoulder width. To minimize external support and ensure consistency, participants were instructed to cross their arms over their chest during the sit-to-stand task. If necessary for safety, they were allowed to rest their hands lightly on their thighs, without pushing or otherwise assisting the movement. A MuscleLab linear encoder (Ergotest Innovation, Norway) was attached to the right posterior waist to record sit-to-stand test data and deliver voice prompts for test initiation and termination in older adults.

6-Min Walking Test (6MWT; Fig. 2A): Participants were instructed to walk back and forth continuously for 6 min at a self-selected pace along a flat, straight walkway that was more than 20 meters long, with clearly marked indicators at both ends. The examiner announced the elapsed time at 2 min intervals. Prior to testing, participants were equipped with both the exosuit and a portable respirometer (COSMED K5, Italy). They stood at one end of the walkway marker. After a 4 min standing rest period for baseline respiratory data collection, the mGait module of the mHealth system (mHealth Technologies, Italy) provided voice prompts to initiate and terminate the test and recorded walking test data.

During the testing period, participants were permitted to reduce their pace or take breaks when necessary, with all pause durations counted into the total testing time. The sequence of experimental conditions (*ExoOff* and *ExoOn*) was randomized for each participant. Within each condition, the order of the functional mobility tests was fixed. To minimize the effects of fatigue, a minimum rest period of 20 min was provided between the two conditions (*ExoOff* and *ExoOn*), and at least 15 min of rest was required between each test (1MSTS and 6MWT).

Data collection was conducted throughout all experiments to evaluate the differences in participants’ performance with and without the exosuit assistance. The IMUs integrated in the exosuit system continuously recorded participants’ kinematic information during all tasks. After completing the experimental tasks under *ExoOn*, participants were asked to complete a ten-item sense-of-agency questionnaire to assess their perceived level of control during exosuit-assisted movements.

#### Data processing and statistics

To evaluate the effectiveness of the exosuit, we assessed sit-to-stand transition efficiency and the metabolic cost of walking in older adults with exosuit assistance. We further examined lower-limb postural stability, kinematics, and users’ sense of agency to determine whether the assistive system maintained stability, preserved natural movement patterns, and perception of being in control over movements in older adults while providing assistance.

During the 1-min sit-to-stand test, we quantified both the total number of completed sit-and-stand cycles and the average time duration required for each cycle as key metrics for evaluating the assistive performance of the exosuit. In addition, the sit-to-stand and stand-to-sit phases were segmented, and the duration of each phase was analyzed and reported separately. The timing data were recorded with a precision of 0.01 s using the MuscleLab system, ensuring accurate temporal resolution for performance analysis.

During the 6-min walking test, the metabolic cost of transport was adopted as the principal evaluation metric to assess the effectiveness of the exosuit in improving walking efficiency. The metabolic cost of transport, which represents the metabolic energy expenditure of walking normalized by body weight and distance, was calculated using the Péronnet and Massicotte equation^39^. Detailed calculation information is provided in the Supplementary Information.

As we described in the Experiment Section, participants were instructed to stand quietly for 4 min to establish a resting metabolic baseline. Considering that metabolic responses undergo a brief adjustment period at the onset of movement as the body transitions from rest to steady-state walking metabolism, the first 2 min of both resting and walking metabolic data were excluded to ensure that only steady-state values were analyzed.

The vertical ground reaction force, vertical center of mass velocity, and center of force data recorded by the force plate system, together with the joint angle and movement velocity captured by the onboard IMU sensors, were analyzed to evaluate the physiological impact of the exosuit on participants’ natural sitting, standing, and walking movements. The collected data were segmented into individual sit-and-stand and walking gait cycles for comparative analysis and intuitive illustration. Kinematic data were assessed by comparing the hip joint and trunk angle, range of motion (ROM), and the positive/negative peak movement velocities across segmented gait cycles. Postural stability was quantified using the 95% confidence ellipse of the center of force trajectory, with the corresponding semi-major and semi-minor axes extracted to characterize sway magnitude.

To evaluate participants’ perceived sense of agency during exosuit-assisted movements, a ten-item questionnaire was administered at the end of the experiment under *ExoOn* condition. The questionnaire was adopted from the study [12]^12^, with items originally derived from the theoretical literature on the sense of agency and further refined for the context of wearable assistive devices^40^. Each item was rated on a 7-point Likert scale ranging from 1 (-Strongly disagree-) to 7 (-Strongly agree-). The questionnaire included six positively framed items (1, 4, 5, 7, 9, 10) and four negatively framed items (2, 3, 6, 8), with negatively framed items inversely coded so that higher scores consistently reflected a stronger sense of agency. After reverse-coding the negatively framed items, a total sense-of-agency score for each participant was obtained by averaging the ten items. In addition, the distribution of participant responses for each questionnaire item was calculated to provide a more detailed understanding of how participants perceived their sense of agency during exosuit-assisted tasks. The mean scores for each questionnaire item across participants were provided in Supplementary Fig. S6.

The normality of data distributions was verified using the Shapiro-Wilk test with a significance level of *α* = 0.05. For variables that satisfied the normality assumption, paired two-tailed t-tests were then performed to compare the results between the *ExoOff* and *ExoOn* conditions. Otherwise, the non-parametric Wilcoxon signed-rank test was applied. For the sense of agency, the overall mean questionnaire score was compared with the scale midpoint of 4 using a two-tailed one-sample t-test. All statistical analyses were conducted using SPSS Statistics (version 27, IBM Corp., USA).

### Reporting summary

Further information on research design is available in the Nature Portfolio Reporting Summary linked to this article.

## Supplementary information

Supplementary Information

Description of Additional Supplementary Files

Supplementary Movie 1

Reporting Summary

Transparent Peer Review file

## Data Availability

The data generated from older adults in this study have been deposited in Figshare under 10.6084/m9.figshare.31315207(ref. ^41^). Supplementary information is available for this paper. Correspondence and requests for materials should be addressed to Xiaohui Zhang.

## Code availability

The exemplary scripts for data processing and analysis for this study have been deposited in an online repository at 10.6084/m9.figshare.31315207(ref. ^41^).
