# Supplementary material for: Enhancing sit-to-stand transitions and walking efficiency in older adults with a soft robotic suit
Source: Nat Commun. 2026 Jul 17;17:6540. doi: 10.1038/s41467-026-75528-1 (PMC13379380; doi:10.1038/s41467-026-75528-1)
Supplement: Supplementary file 1 — Supplementary Information [file 41467_2026_75528_MOESM1_ESM.pdf]

Enhancing sit-to-stand transitions and walking efficiency in older adults with a soft robotic suit

Xiaohui Zhang[†1*], Enrica Tricomi[†1], Marios Stefanakis[2], Nathalie Gierden[1], Theresa Buchner[2], Luka Mišković[1], Jürgen M. Bauer[2,3], Christian Werner[2], Clemens Becker[2,3] and Lorenzo Masia[1]

[1]Department of Computer Engineering, School of Computation, Information and Technology, Technical University of Munich, Munich, Germany.
[2]Geriatric Center, Medical Faculty Heidelberg, Heidelberg University, Heidelberg, Germany.
[3]Network Aging Research, Heidelberg University, Heidelberg, Germany.

*Corresponding author. E-mail: xiaohui.zhang@tum.de;
Contributing authors: † These authors contributed equally to this work;

SUPPLEMENTARY INFORMATION

This PDF file includes

- Hardware Components and Locations
- Controller Implementation
- Individual Sit-to-Stand Performance
- Individual Walking Performance
- Individual Gait Smoothness
- Distribution of Sense of Agency Responses
- Effect of Exosuit Assistance Compared with No-Exosuit Condition

Other Supplementary Material

- Recorded data and code for analyses
- Video accompanying the paper

Hardware Components and Locations

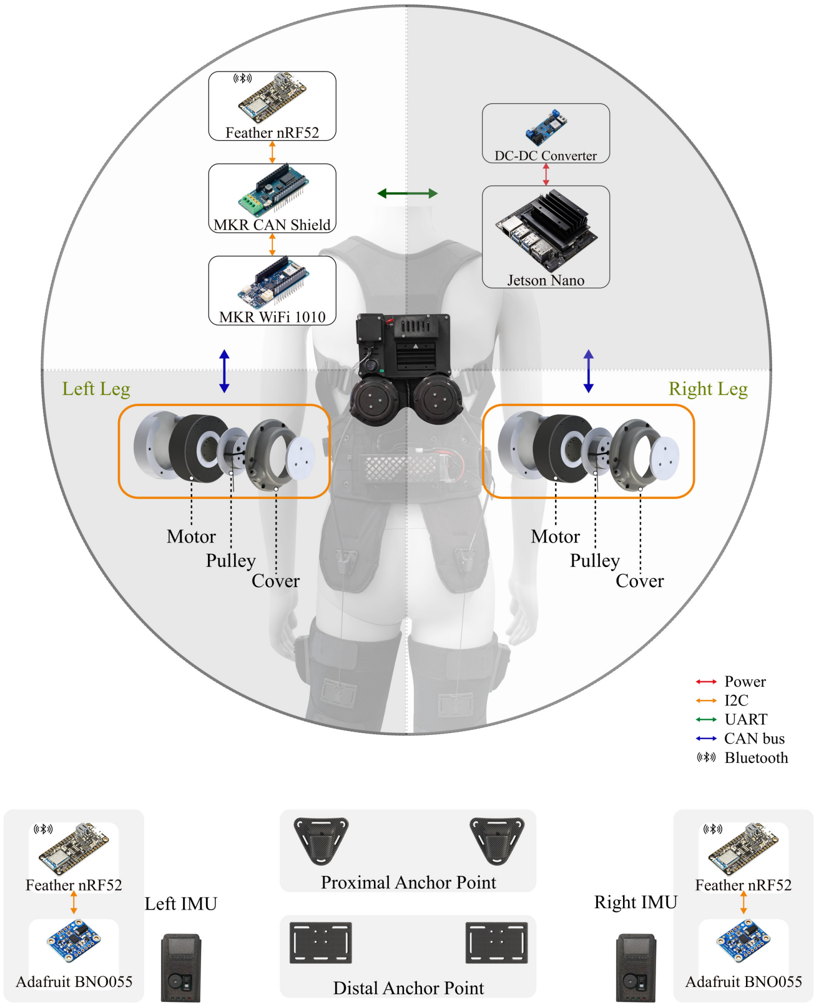

Fig. S1 Overview of the hardware components and locations of the soft wearable exosuit. The main components of the soft wearable exosuit include the onboard electronics, actuation modules, and sensing units. During operation, the sensing units (left and right IMUs) detect lower-limb motion and transmit sensor information to the onboard control electronics. The control system then estimates the user's locomotion state and generates task-specific assistance commands. The actuation modules subsequently deliver assistive forces to the user through the proximal and distal anchor points. Coloured arrows indicate the main electrical and communication connections.

In the soft wearable exosuit, the back-mounted hardware box, together with the left and right IMU units integrates all key electronics. Fig. S1 illustrates

the detailed electronic components within each module. IMU units based on the Adafruit BNO055 (Bosch, BNO055, Germany) are mounted on the left and right thighs to measure the user's hip joint angle and velocity. These kinematic signals are transmitted in real time via Bluetooth from a Feather board (BLE, Feather nRF52 Bluefruit, Adafruit, USA) to a second Feather board located in the upper-left corner of the back hardware enclosure. The signals are then forwarded via the Inter-Integrated Circuit (I^2C) bus to an Arduino (MKR 1010 WiFi, Arduino, Italy) equipped with a CAN-bus shield (CAN-bus Shield V2.0, Seeed Studio, China). Finally, the processed data are transmitted in real time to the Jetson Nano (NVIDIA, USA) for locomotion mode detection and gait phase estimation. The Jetson generates motor reference trajectories that are sent back to the Arduino, which drives the actuators via the Can-bus, rotating the motors and the pulleys mounted above them. Rotation of these pulleys tightens and releases the artificial tendons routed to proximal and distal anchor points on each thigh, thereby delivering assistive forces to the user. In addition to the electronic boards, the housings, pulley components, and both proximal and distal anchor points are fabricated by 3D printing technology with PLA (polylactic acid).

Controller Implementation

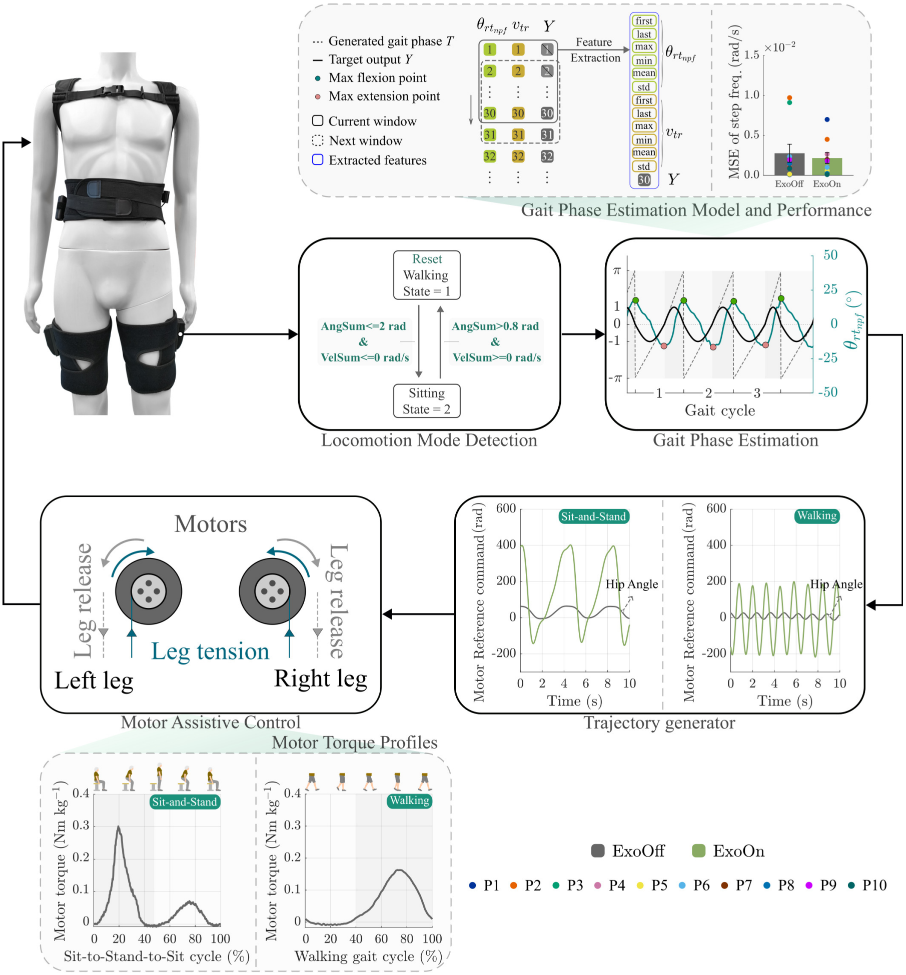

Fig. S2 Schematic diagram and detailed illustration of the controller's working principle. The controller processes thigh kinematic signals to detect the locomotion mode and estimate the gait phase in real time. The detected locomotion mode and estimated gait phase are then used by the trajectory generator to produce task-specific motor reference commands for sit-to-stand transitions and walking. These commands are executed by the bilateral motor assistive control module, which regulates tendon release and leg tension to deliver assistance to the user's left and right legs. Representative motor reference commands and motor torque profiles for sit-and-sit and walking cycles are shown as examples.

High Level of the Controller

Locomotion mode detection: The exosuit provides assistive forces to the

user during sit-to-stand transitions and leg extension in walking. Locomotion mode detection of the exosuit is implemented using a finite-state machine that switches between sitting and walking modes based on threshold conditions on the bilateral hip joint angle (AngSum) and velocity (VelSum). Fig. S2 also presents the motor torque profiles from a representative participant during sit-to-stand and overground walking movements. Compared with walking, the magnitude of assistance delivered during sit-to-stand transfers is higher.

Gait phase estimation model: Eight healthy participants were recruited for data collection for the gait phase estimation model. Each participant completed a 400m walk on level ground at a self-selected speed followed by one minute of quiet standing. Two kinematic signals were selected as input for the gait phase estimation model: the notch-peak filtered angle ($\theta_{rt_{npf}}$) and the angular velocity (v_{rt}) of the thigh. These signals reflect key dynamic characteristics throughout the gait cycle and were acquired in real time within a MATLAB/Simulink environment. The output labels for model training were generated through a semi-manual annotation process. As illustrated in Fig. S2, maximum flexion points and maximum extension points were detected from the $\theta_{rt_{npf}}$ signal to segment individual gait cycles. For each cycle, a continuous gait phase vector T was constructed by linearly interpolating values from $[-\pi, 0]$ between the maximum flexion point and the maximum extension point, and from $[0, \pi]$ between the maximum extension point and the subsequent maximum flexion point. To ensure phase continuity across cycles and avoid discontinuities at transitions, the final target output was defined as $Y = \sin(T + \tau)$, with $\tau \in [0, \pi]$ representing a tunable delay compensation parameter initialized to 0.

Here, we obtained two kinematic input signals and one output label Y to construct the training dataset for the regression model. A sliding window technique was applied to the raw sequential signals to extract gait phase features. At each sliding step (corresponding to one sample interval at a sampling rate of 100 Hz in the MATLAB/Simulink environment), the window captured 30 consecutive samples from each signal channel. For each input signal within the current window (e.g., $\theta_{rt_{npf}}$ and v_{rt}), six statistical features were computed: the first value, last value, maximum, minimum, mean, and standard deviation. These features were concatenated across the two channels to form a 12-dimensional input feature vector. The corresponding label for each feature vector was defined as the last value of the gait phase label Y within the same window. The regression model was trained using the *fitrensemble* function in MATLAB/Simulink R2021b, incorporating 30 decision trees with a maximum of 1024 splits each. The final prediction was derived by averaging the outputs of the individual trees.

Since the gait phase estimation model was trained on data from healthy young participants, we evaluated its gait phase estimation performance in older adults by quantifying the mean absolute error (MAE) between the actual and estimated gait frequency during walking across all participants. The MAE result of gait frequency estimation was less than 0.003 for all participants (n = 10) in both *ExoOff* and *ExoOn* conditions. These results demonstrate the effectiveness of the gait phase estimation model across different age groups.

Low-Level of the Controller The trajectory generator uses the estimated gait phase Y from the high-level of the controller to produce appropriate reference trajectories for different locomotion modes. Fig. S2 shows the motor reference trajectories for a representative participant across the different modes. Specifically, in walking mode, the predicted gait phase Y is scaled

by a gain factor $k \in [1, 5]$ to generate the target motor trajectory, expressed as $R_{traj} = k * Y$. The scaling factor k can be flexibly adjusted according to the individual user's needs to achieve personalized assistance. In sit-to-stand transitions, the controller enhances actuator responsiveness and vertical support by using the right leg's angular velocity v_{rt} as the control variable to define the reference trajectory as $R_{traj} = 2 * v_{rt}$. To ensure smooth transitions between modes and to avoid abrupt actuator responses, the reference trajectory is passed through a second-order low-pass filter with a cutoff frequency of 4.8 Hz. This filtering effectively attenuates high-frequency noise and ensures the continuity and stability of the generated motion profiles.

The filtered reference trajectory is then tracked by a proportional controller with a first-order low-pass characteristic. This controller converts the error between the desired and actual motor positions into a motor angular velocity command. Its transfer function is given by:

$$H_p(s) = \frac{K_p}{1 + K_d \cdot s} \tag{1}$$

where $K_p = 8$ and $K_d = 0.06$ are experimentally tuned parameters that were found to provide a good balance between fast system response, tracking accuracy, and user comfort.

Individual Sit-to-Stand Performance

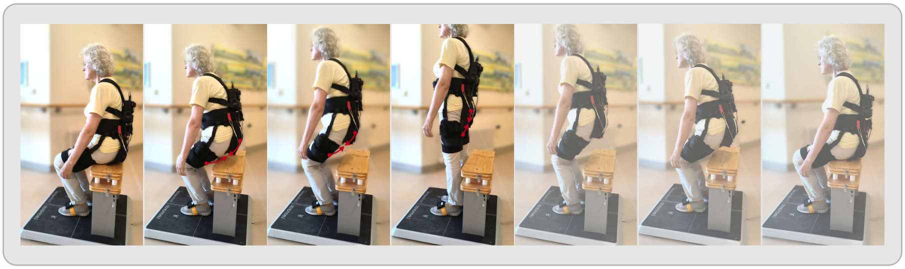

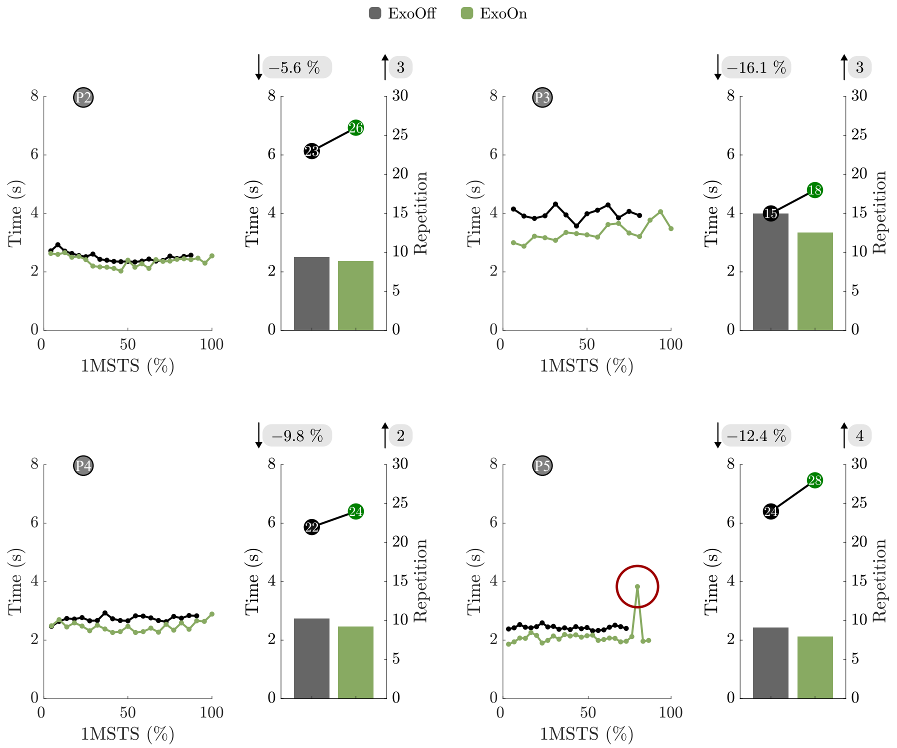

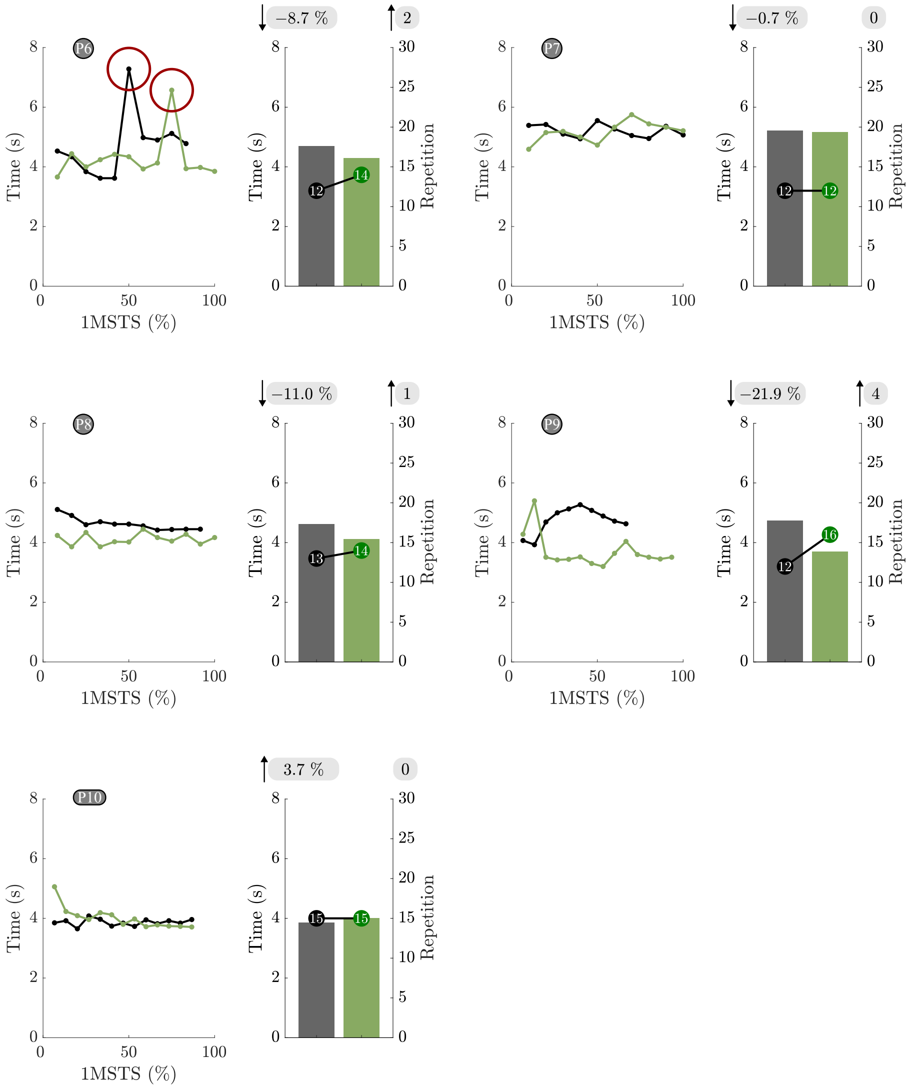

Fig. S3 1-Minute Sit-to-Stand Test: individual sit-and-stand duration, mean duration, and total repetition under *ExoOff* and *ExoOn* conditions. The line plot on the left for each participant shows the time duration of each sit-and-stand repetition during the 1-minute sit-to-stand test (1MSTS), with each point representing the duration of a single sit-and-stand movement. The right panel (bars) shows the mean sit-and-stand time within the 1MSTS together with the total number of repetitions (circles with numbers). Grey denotes *ExoOff* condition and green denotes *ExoOn* condition.

Across the nine older adults who performed the 1-minute sit-to-stand test (1MSTS, n = 9, Fig. S3), exosuit assistance generally improved sit-to-stand performance. Two participants completed four additional sit-and-stand cycles within 1 minute in the *ExoOn* condition compared with *ExoOff*, with their mean time per cycle reduced by 21.9% and 12.4%, respectively. Two other participants completed three additional repetitions, two completed two additional repetitions, and one completed one additional repetition, whereas the remaining two participants performed the same number of repetitions with and without assistance. In Fig. S3, red circles highlight individual sit-and-stand cycles with a sudden increase in duration, indicating brief rests taken by the older participants. For example, in participant 6 (P6), these prolonged cycles occur noticeably earlier in the *ExoOff* condition than in the *ExoOn* condition, suggesting that the exosuit assistance delays the onset of fatigue and enables more consistent sit-to-stand performance for this participant.

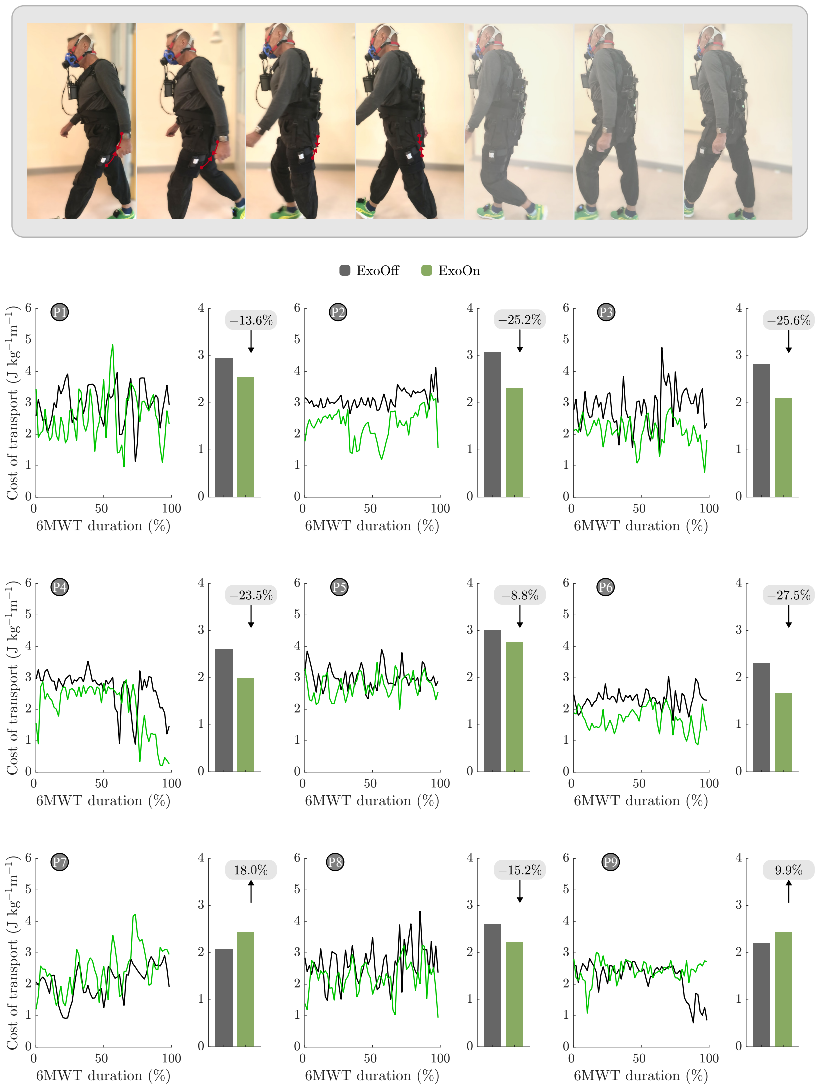

Fig. S4 6-Minute Walking Test: individual metabolic cost of transport under *ExoOff* (grey) and *ExoOn* (green) conditions. The line plot on the left illustrates the steady-state metabolic cost of transport for each participant throughout the 6-minute walking test. The bar on the right shows the corresponding mean steady-state metabolic cost.

During the 6-minute walking test, the metabolic cost of transport (C) was calculated as

$$C = \frac{P - P_0}{w * v} \tag{2}$$

where P is the participant's metabolic cost during the 6-minute walking test, P_0 is the baseline metabolic cost measured at rest, w denotes the body weight, and v represents the average walking speed, which was acquired through the mGait module within the mHealth system. The metabolic cost P was calculated using the Péronnet and Massicotte equation (Ref. [39] in the main manuscript):

$$P = 16.89V_{O_2} + 4.84V_{CO_2} \tag{3}$$

here, V_{O_2} and V_{CO_2} correspond to the rates of oxygen consumption and carbon dioxide production, respectively, which were continuously measured breath-by-breath during walking using a portable respiratory system.

Individual walking metabolic cost of transport information of older adults was shown in Fig. S4. Across the nine older adults who completed the 6-minute walking test (n = 9), 7 out of 9 (77.8%) demonstrated a reduction in metabolic cost of transport when walking with exosuit assistance compared with the *ExoOff* condition. This indicates that the exosuit delivered a beneficial metabolic effect for the majority of older adults. Two participants instead showed increased metabolic expenditure during *ExoOn*, including one with an 18% increase. These findings underscore the substantial heterogeneity in how older adults respond to exosuit mechanical assistance. The observed increases in metabolic cost may reflect limited adaptation to the exosuit, which can induce anxiety or prompt compensatory gait strategies that elevate energetic demand.

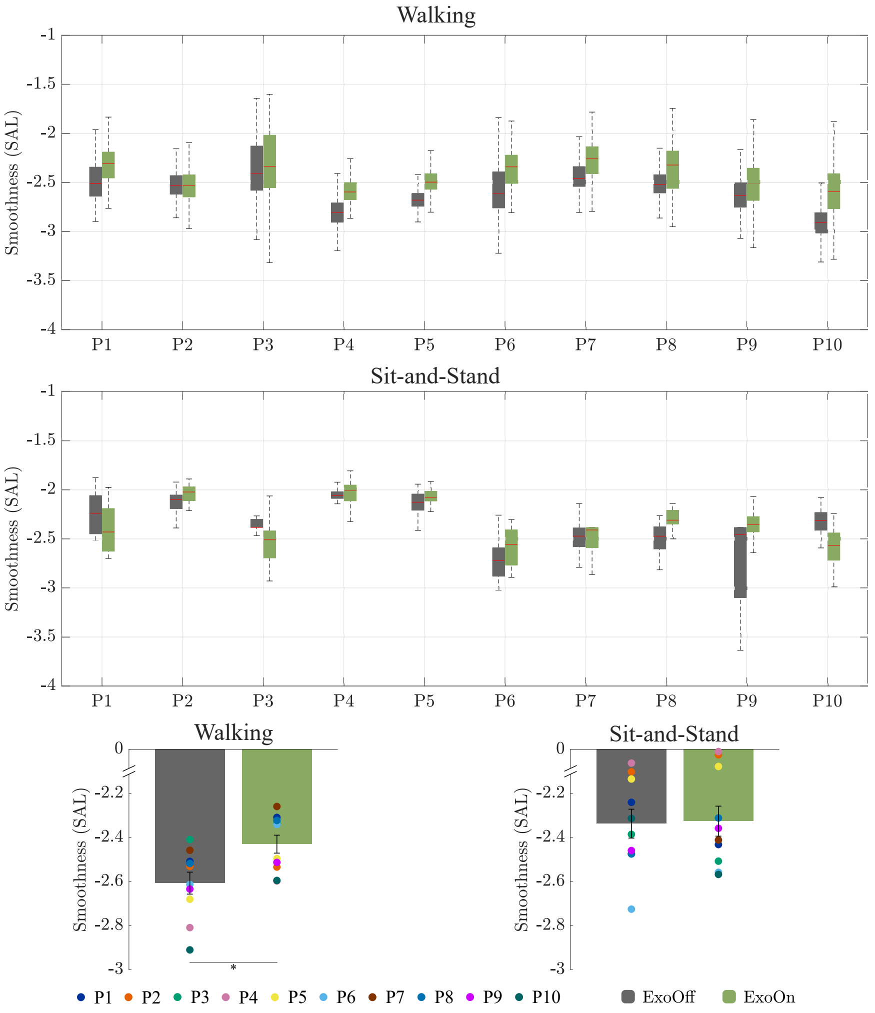

Fig. S5 Individual and mean gait Smoothness in Spectral Arc Length (SPARC) under *ExoOff* and *ExoOn* conditions. The central red line in each box indicates the individual median SPARC computed over all segmented steps during walking or over all sit-to-stand repetitions, and the lower and upper edges of each box represent the 25th and 75th percentiles. The bars shown below represent the mean (± s.e.m., n = 10) smoothness (SPARC) across participants. * indicates statistically significant differences ($p < 0.05$, paired two-tailed t-tests).

The boxplots (Fig. S5) indicate that participants exhibited significantly ($n = 10$, $p < 0.001$) lower Spectral Arc Length (SPARC) values during walking in the *ExoOn* condition than in *ExoOff* (2.60 ± 0.05, 2.43 ± 0.04, respectively, mean $\pm$ s.e.m.), reflecting smoother movement with assistance. In contrast, overall smoothness during the sit-to-stand was lower than during walking, likely because this task places greater demands on balance control for older adults. Marked inter-individual variability was also observed in sit-to-stand smoothness. While seven participants showed higher or comparable smoothness under *ExoOn*, three exhibited reduced smoothness with assistance. This suggests that exosuit assistance may disrupt the natural timing of the transition for some individuals, or that difficulty adapting to the device may induce anxiety, increase effort, and elicit compensatory movement strategies, ultimately reducing smoothness.

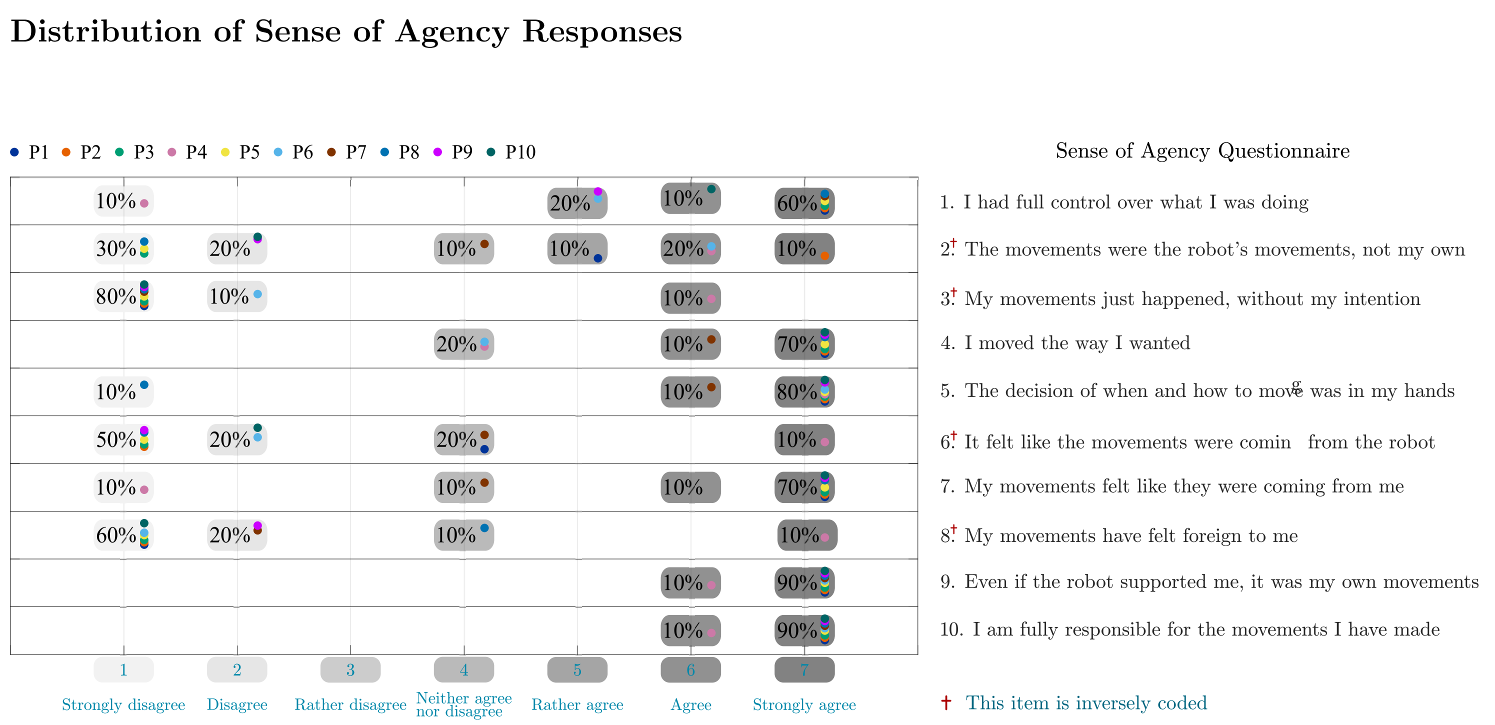

Fig. S6 The percentage distribution of responses to the 10-item sense of agency questionnaire (n = 10). Participants' responses to each item of the 7-point sense of agency questionnaire (ranging from "Strongly disagree" to "Strongly agree"). For each question, the percentage labels represent the proportion of participants selecting each Likert response option, while colored circular markers denote individual participants' responses. Items 2, 3, 6, and 8 are inversely coded, whereas items 1, 4, 5, 7, 9, and 10 follow the standard scoring direction.

Effect of Exosuit Assistance Compared with No-Exosuit Condition

Participants: To provide a baseline without wearing the exosuit (*NoExo*), a further experimental session was conducted in a subgroup of 7 of the 10 originally enrolled participants (3 females and 4 males; age 69–85 years, mean 79.0 ± 6.0 years; height 171.7 ± 5.0 cm; weight 66.3 ± 3.4 kg, mean ± SD).

In this supplementary experiment, participants repeated the same protocol (1-minute sit-to-stand test and 6-minute walking test) described in the main manuscript without wearing the exosuit, while retaining only the recording unit with IMU sensors and the metabolic analyzer to enable kinematic and metabolic recordings and comparisons. Accordingly, comparisons among *NoExo* and *ExoOn* conditions reported in this section were performed within this subgroup.

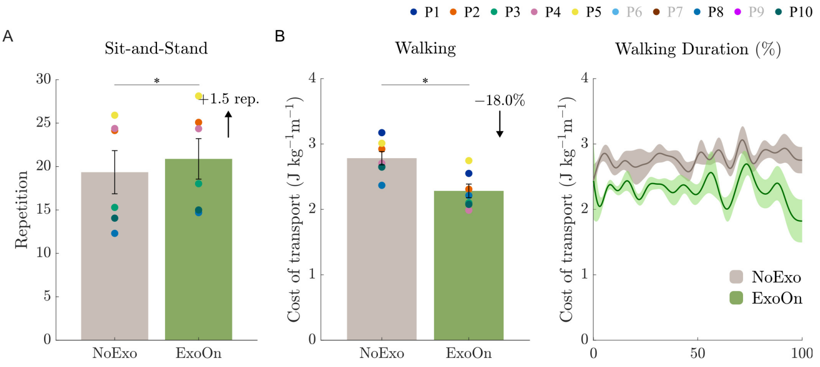

Fig. S7 Performance evaluation of the exosuit in older adults under *NoExo* and *ExoOn* conditions. A Total repetitions in 1-minute sit-to-stand test (n = 6). **B** Steady Walking metabolic cost of transport measured during the 6-minute walking test (n = 7). Bars and curves indicate mean ± s.e.m., with light brown and green bars denoting the *NoExo* and *ExoOn* conditions, respectively. Dots represent individual participants, with bold black labels indicating the 7 participants included in this supplementary analysis and grey-shaded labels indicating originally enrolled participants who were unavailable and therefore not included. * denotes a statistically significant difference ($p < 0.05$, paired two-tailed t-tests).

Sit-to-stand efficiency was found to be significantly improved in older adults. The comparison between the *NoExo* and *ExoOn* conditions

indicated that exosuit assistance enhanced sit-to-stand transition performance. During the 1-minute sit-to-stand test, participants completed 1.5 ± 0.4 more cycles in the *ExoOn* condition than in the *NoExo* condition (19.4 ± 2.5 repetitions in *NoExo* and 20.9 ± 2.3 repetitions in *ExoOn*; n = 6, p = 0.017; Fig. S7A, left). Exosuit assistance also shortened sit-and-stand cycle duration, with a maximum individual reduction of 16.3% (4.87 s in *NoExo* and 4.08 s in *ExoOn*). Participant 1 (P1) was also excluded from the comparison between *NoExo* and *ExoOn* in the sit-to-stand analysis because only 30 s of sit-to-stand data were available.

Metabolic cost of transport during walking was significantly reduced with exosuit assistance. Exosuit assistance significantly reduced the metabolic cost of transport in older adults, with a mean reduction of 18.0% across participants (from 2.78 ± 0.10 $Jkg^{-1}m^{-1}$ to 2.28 ± 0.10 $Jkg^{-1}m^{-1}$; n = 7, $p < 0.001$) (Fig. S7B). All participants exhibited a lower metabolic cost of transport with exosuit assistance. The largest individual reduction reached 26.5%, decreasing from 2.70 $Jkg^{-1}m^{-1}$ to 1.99 $Jkg^{-1}m^{-1}$.

Lower-limb natural kinematics and stability were largely preserved. Lower-limb kinematics and stability-related measures during sit-to-stand and walking tasks were also analyzed and compared between *NoExo* and *ExoOn* conditions to assess potential alterations in natural movement associated with exosuit assistance. Across both sit-to-stand and walking tasks, hip joint kinematics remained broadly comparable with exosuit assistance. During the sit-to-stand task (Fig. S8A), hip angle range of motion changed from 69.08 ± 3.07 ° in the *NoExo* condition to 67.74 ± 1.91 ° in the *ExoOn* condition. Hip peak velocity changed from 83.66 ± 8.40 $°s^{-1}$ to 78.11 ± 7.85 $°s^{-1}$ for the positive peak, and from −93.28 ± 5.60 $°s^{-1}$ to −90.87 ± 5.00 $°s^{-1}$ for the negative peak. During walking (Fig. S8B), hip angle range of motion was significantly

different between the *NoExo* and *ExoOn* conditions, changing from 40.81 ± 1.34 ° to 38.52 ± 1.56 ° (n = 7, $p < 0.001$). Hip peak velocity changed from 161.78 ± 9.44 $°s^{-1}$ to 154.05 ± 6.62 $°s^{-1}$ for the positive peak, whereas the negative peak velocity was also significantly different, changing from −102.80 ± 6.52 $°s^{-1}$ to −93.05 ± 7.02 $°s^{-1}$ (n = 7, p = 0.021).

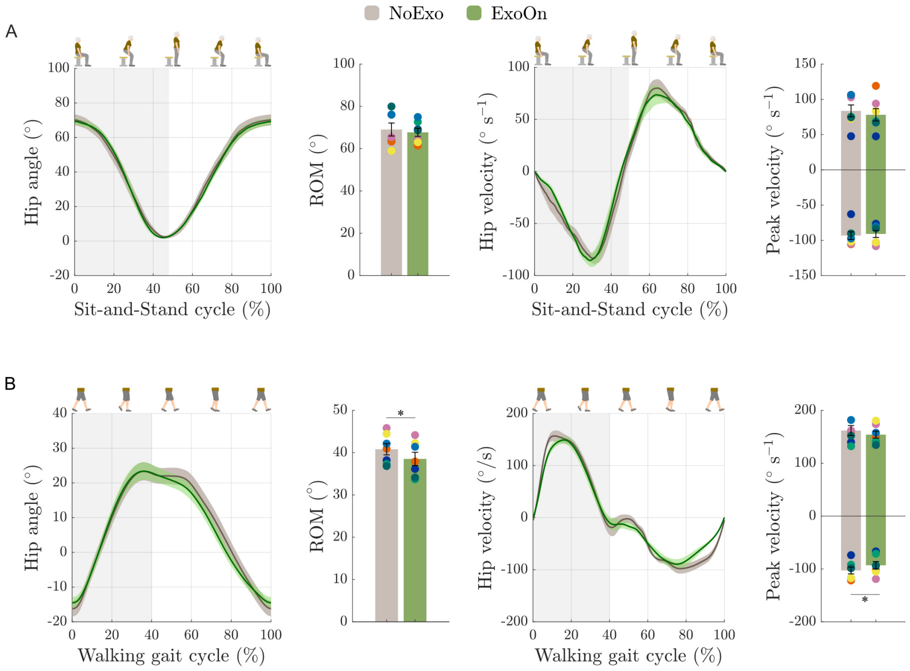

Fig. S8 Kinematic evaluation in older adults under *NoExo* and *ExoOn* conditions (n = 7). Hip angle and velocity profile during **A** sit-to-stand and **B** walking movements. Range of motion (ROM) was calculated as the difference between the maximum and minimum hip angles within each segmented sit-and-stand or walking cycle across steps and participants. Velocity profiles were quantified by peak positive and peak negative velocity values. Bar and curve plots show the mean ± s.e.m. for the *NoExo* (light brown) and *ExoOn* (green) conditions across all the participants. * indicates statistically significant differences ($p < 0.05$, paired two-tailed t-tests).

The 95% confidence ellipse of the centre-of-force (COF) trajectory measured from the ground reaction force platform was further analyzed, and no significant differences were observed between the *NoExo* and *ExoOn* conditions (n = 6). As shown in Fig. S9A, the COF ellipse area was 1.58 ± 0.37 cm^2

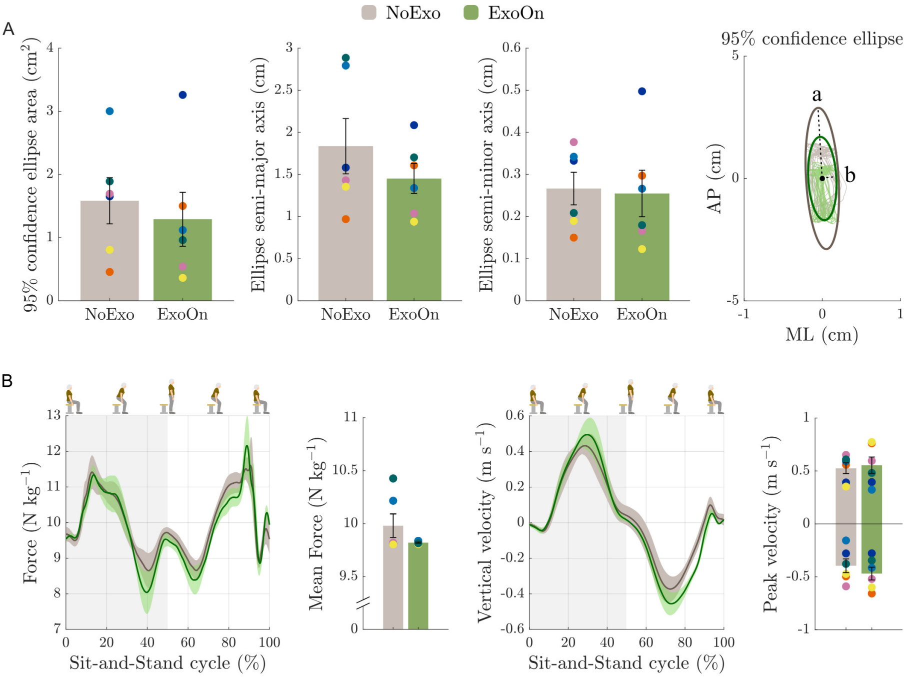

Fig. S9 Biomechanical analysis and stability assessment of sit-to-stand movement in older adults under *NoExo* and *ExoOn* conditions (n = 6). **A** 95% confidence ellipse fitted to centre of force (COF) data points across participants during sit-to-stand test. The lengths of the semi-major axis (a) and semi-minor axis (b) also served as metrics for quantifying postural sway of the plantar pressure in the anteroposterior (AP) and mediolateral (ML) directions, respectively. Data from a representative participant are shown for illustration, with AP and ML coordinates zero-centered. **B** Trajectory and mean vertical ground reaction force and velocity across older participants. Bar and curve plots show mean ± s.e.m.; dots indicate individual participants.

in *NoExo* and 1.29 ± 0.43 cm^2 in *ExoOn*. The semi-major axis length in the AP direction was 1.84 ± 0.33 cm and 1.45 ± 0.18 cm, whereas the minor axis length in the ML direction was 0.27 ± 0.04 cm and 0.25 ± 0.06 cm, respectively. In addition, the total ground reaction force and centre-of-mass velocity profiles (Fig. S9B) were similar across the two conditions. Mean ground reaction force was 9.98 ± 0.11 Nkg^{-1} in *NoExo* and 9.82 ± 0.005 Nkg^{-1} in *ExoOn*. Peak centre of mass velocity also showed only small differences, with maximum values of 0.53 ± 0.05 ms^{-1} and 0.55 ± 0.08 ms^{-1} and minimum values of

-0.40 ± 0.06 ms^{-1} and -0.47 ± 0.06 ms^{-1} in the *NoExo* and *ExoOn* conditions, respectively. Participant 3 (P3) was excluded from the stability analysis due to data missing.

Conclusion: Results from the *NoExo* and *ExoOn* conditions showed trends broadly consistent with those observed in the *ExoOff* and *ExoOn* analysis reported in the main manuscript. Compared with *NoExo*, exosuit assistance (*ExoOn*) significantly increased 1-minute sit-to-stand performance by an average of 1.5 repetitions across all the participants (Fig. S7A; $p = 0.017$), compared with the average increase of 1.8 repetitions observed relative to the *ExoOff* condition (Fig. 2B in the main manuscript, $p = 0.006$). During walking, exosuit assistance significantly reduced the metabolic cost of transport by 18.0% (Fig. S7B; $p < 0.001$), compared with the 13.6% reduction observed relative to the *ExoOff* condition in the main manuscript (Fig. 2D; $p = 0.030$). These findings suggest that exosuit assistance compensated for the burden associated with wearing the exosuit in the *ExoOff* condition and provided a measurable net energetic benefit during sustained walking. In addition, exosuit assistance was associated with significant changes only in walking hip range of motion and negative peak angular velocity. However, the overall lower-limb movement pattern remained largely preserved, and force-related stability metrics were maintained (Fig. S8, S9). These findings further support the conclusion that the soft exosuit enhanced sit-to-stand and walking performance in older adults without substantially altering movement kinematics or compromising stability.
