# Supplementary material for: Enhancing sit-to-stand transitions and walking efficiency in older adults with a soft robotic suit
Source: Nat Commun. 2026 Jul 17;17:6540. doi: 10.1038/s41467-026-75528-1 (PMC13379380; doi:10.1038/s41467-026-75528-1)
Supplement: Supplementary file 2 — Description of Additional Supplementary Files [file 41467_2026_75528_MOESM2_ESM.pdf]

Description of Additional Supplementary Files

File name: Supplementary Movie 1
Description: This movie summarizes this work, including research background, experimental design, and main conclusions.
