# Supplementary material for: Enhancing sit-to-stand transitions and walking efficiency in older adults with a soft robotic suit
Source: Nat Commun. 2026 Jul 17;17:6540. doi: 10.1038/s41467-026-75528-1 (PMC13379380; doi:10.1038/s41467-026-75528-1)
Supplement: Supplementary file 4 — Reporting Summary [file 41467_2026_75528_MOESM4_ESM.pdf]

nature portfolio

Corresponding author(s): Xiaohui Zhang

Last updated by author(s): May 26, 2026

Reporting Summary

Nature Portfolio wishes to improve the reproducibility of the work that we publish. This form provides structure for consistency and transparency in reporting. For further information on Nature Portfolio policies, see our Editorial Policies and the Editorial Policy Checklist.

Statistics

For all statistical analyses, confirm that the following items are present in the figure legend, table legend, main text, or Methods section.

n/a	Confirmed	
☐	☒	The exact sample size (*n*) for each experimental group/condition, given as a discrete number and unit of measurement
☐	☒	A statement on whether measurements were taken from distinct samples or whether the same sample was measured repeatedly
☐	☒	The statistical test(s) used AND whether they are one- or two-sided<br>*Only common tests should be described solely by name; describe more complex techniques in the Methods section.*
☒	☐	A description of all covariates tested
☐	☒	A description of any assumptions or corrections, such as tests of normality and adjustment for multiple comparisons
☐	☒	A full description of the statistical parameters including central tendency (e.g. means) or other basic estimates (e.g. regression coefficient) AND variation (e.g. standard deviation) or associated estimates of uncertainty (e.g. confidence intervals)
☐	☒	For null hypothesis testing, the test statistic (e.g. *F*, *t*, *r*) with confidence intervals, effect sizes, degrees of freedom and *P* value noted<br>*Give P values as exact values whenever suitable.*
☒	☐	For Bayesian analysis, information on the choice of priors and Markov chain Monte Carlo settings
☒	☐	For hierarchical and complex designs, identification of the appropriate level for tests and full reporting of outcomes
☒	☐	Estimates of effect sizes (e.g. Cohen's *d*, Pearson's *r*), indicating how they were calculated

Our web collection on statistics for biologists contains articles on many of the points above.

Software and code

Policy information about availability of computer code

Data collection	Kinematic data from the inertial measurement units (IMUs) were collected using MATLAB/Simulink (R2021b). Sit-and-stand repetition data during the 1-minute sit-to-stand test were recorded using the MuscleLab system (MuscleLab 23). Walking speed data during the 6-minute walking test were recorded using the mGait module of the mHealth system (version 1.0). Ground reaction force data were acquired using a Leonardo Mechanography force platform and recorded with Leonardo Mechanography Basic Edition software (version 4.4b02.9). Respiratory data were recorded using a COSMED K5 portable metabolic system with OMNIA software (version 1.6).
Data analysis	Data processing and analysis were performed in MATLAB (R2021b).<br>Statistical analyses were performed using IBM SPSS Statistics 27.

For manuscripts utilizing custom algorithms or software that are central to the research but not yet described in published literature, software must be made available to editors and reviewers. We strongly encourage code deposition in a community repository (e.g. GitHub). See the Nature Portfolio guidelines for submitting code & software for further information.

Data

Policy information about availability of data

All manuscripts must include a data availability statement. This statement should provide the following information, where applicable:

- Accession codes, unique identifiers, or web links for publicly available datasets
- A description of any restrictions on data availability
- For clinical datasets or third party data, please ensure that the statement adheres to our policy

Data necessary to replicate this work have been deposited in an online repository at https://doi.org/10.6084/m9.figshare.31315207

Research involving human participants, their data, or biological material

Policy information about studies with human participants or human data. See also policy information about sex, gender (identity/presentation), and sexual orientation and race, ethnicity and racism.

Reporting on sex and gender	The main experiment enrolled ten older adults (n=10) who completed the ExoOff and ExoOn conditions, comprising six females and four males.<br>The supplementary experiment enrolled a subgroup of seven older adults (n=7) who completed the NoExo and ExoOn conditions, comprising three females and four males.
Reporting on race, ethnicity, or other socially relevant groupings	Race, ethnicity, and other socially relevant characteristics were not collected or used for participant grouping, as they were not considered relevant to the outcomes of this study.<br>Participants were selected based on age-related eligibility and functional criteria.
Population characteristics	Ten older adults participants (n=10) were recruited in the ExoOff and ExoOn conditions (six females and four males; age range, 69–85 years; age, 77.7 ± 6.1 years; height, 169.3 ± 6.9 cm; weight, 69.1 ± 9.2 kg; mean ± SD).<br>Seven older adults participants (n=7) from the original cohort were enrolled in the NoExo and ExoOn conditions (three females and four males; age range, 69–85 years; age, 79.0 ± 6.0 years; height, 171.7 ± 5.0 cm; weight, 66.3 ± 3.4 kg; mean ± SD).
Recruitment	Community-dwelling older adults at risk of functional decline (n = 10) were recruited through the research department of Bethanien Hospital Heidelberg, Germany. Eligible participants were over 65 years of age and able to stand up from a chair at least once without using their arms, as assessed by the single chair-stand test. Exclusion criteria included conditions that could compromise safe participation, such as severe uncorrected visual or auditory impairments, significant neurological, cardiovascular, metabolic, or mental disorders, cognitive impairment (Mini-Mental State Examination, MMSE ≤ 24), acute musculoskeletal pain during sit-to-stand, or unstable cardiopulmonary disease.
Ethics oversight	Research procedures were approved by the Ethics Committee of Heidelberg University under resolution No. S-313/2020.

Note that full information on the approval of the study protocol must also be provided in the manuscript.

Field-specific reporting

Please select the one below that is the best fit for your research. If you are not sure, read the appropriate sections before making your selection.

☒ Life sciences ☐ Behavioural & social sciences ☐ Ecological, evolutionary & environmental sciences

For a reference copy of the document with all sections, see nature.com/documents/nr-reporting-summary-flat.pdf

Life sciences study design

All studies must disclose on these points even when the disclosure is negative.

Sample size	The sample size was chosen based on participant eligibility, availability, practical constraints, and the feasibility-focused nature of the study. Ten older adults were recruited for the ExoOff and ExoOn conditions, and seven participants from the original cohort were re-enrolled for the NoExo and ExoOn conditions. These sample sizes are consistent with related wearable lower-limb assistive-device studies (Haufe, F. L. et al. 2021; Tricomi, E. et al. 2024), which commonly include more than six participants.
Data exclusions	No exclusions were made.
Replication	All experimental data and exemplary scripts for data analysis are available to replicate the results shown in the Figures of the manuscript.
Randomization	The sequence of experimental conditions (ExoOff/NoExo and ExoOn) was randomized for each participant. Within each condition, the order of the functional mobility tests was fixed (1. 1MSTS; 2. 6MWT).<br>To minimize the effects of fatigue, a minimum rest period of 20 minutes was provided between the two conditions (ExoOff/NoExo and ExoOn) and at least 15 minutes of rest was required between each test (1. 1MSTS; 2. 6MWT).

Blinding

Blinding was not relevant to the study because the participant could readily recognize whether they were wearing the exosuit and whether they were moving unassisted or receiving assistance from the soft robotic shorts.

Reporting for specific materials, systems and methods

We require information from authors about some types of materials, experimental systems and methods used in many studies. Here, indicate whether each material, system or method listed is relevant to your study. If you are not sure if a list item applies to your research, read the appropriate section before selecting a response.

Materials & experimental systems

n/a	Involved in the study
☒	☐ Antibodies
☒	☐ Eukaryotic cell lines
☒	☐ Palaeontology and archaeology
☒	☐ Animals and other organisms
☒	☐ Clinical data
☒	☐ Dual use research of concern
☒	☐ Plants

Methods

n/a	Involved in the study
☒	☐ ChIP-seq
☒	☐ Flow cytometry
☒	☐ MRI-based neuroimaging

Plants

Seed stocks

Report on the source of all seed stocks or other plant material used. If applicable, state the seed stock centre and catalogue number. If plant specimens were collected from the field, describe the collection location, date and sampling procedures.

Novel plant genotypes

Describe the methods by which all novel plant genotypes were produced. This includes those generated by transgenic approaches, gene editing, chemical/radiation-based mutagenesis and hybridization. For transgenic lines, describe the transformation method, the number of independent lines analyzed and the generation upon which experiments were performed. For gene-edited lines, describe the editor used, the endogenous sequence targeted for editing, the targeting guide RNA sequence (if applicable) and how the editor was applied.

Authentication

Describe any authentication procedures for each seed stock used or novel genotype generated. Describe any experiments used to assess the effect of a mutation and, where applicable, how potential secondary effects (e.g. second site T-DNA insertions, mosiacism, off-target gene editing) were examined.
