# Supplementary material for: Enhancing sit-to-stand transitions and walking efficiency in older adults with a soft robotic suit
Source: Nat Commun. 2026 Jul 17;17:6540. doi: 10.1038/s41467-026-75528-1 (PMC13379380; doi:10.1038/s41467-026-75528-1)
Supplement: Supplementary file 5 — Transparent Peer Review file [file 41467_2026_75528_MOESM5_ESM.pdf]

nature portfolio

Peer Review File

Enhancing sit-to-stand transitions and walking efficiency in older adults with a soft robotic suit

Corresponding Author: Dr Xiaohui Zhang

This file contains all reviewer reports in order by version, followed by all author rebuttals in order by version.

Version 0:

Reviewer comments:

Reviewer #1

(Remarks to the Author)
In this paper the authors set forth present a soft tendon-driven hip exosuit designed to support sit-to-stand transition (STST) and walking for older adults with reduced lower-limb functional performance. Although this is a well written paper and on an interesting area that is within the scope of the journal further revisions are required ahead of publication.
-The manuscript reports improved STST, but it remains unclear how the exosuit produces this benefit from a biomechanical perspective. STST begins from a highly flexed posture in which the lower limb operates with poor leverage, large joint ranges of motion, and likely reduced effective mechanical advantage (EMA), especially in the early phase and around seat-off. I therefore encourage the authors to clarify whether they assessed phase-specific joint mechanics during STS and a discussion of posture-dependent leverage and EMA would substantially strengthen the mechanistic interpretation.
-Authors present benefits for both STST and walking, but these two tasks differ fundamentally in mechanical and neuromuscular demands. Walking often benefits from near-isometric muscle action and elastic tendon behavior during stance, whereas STST requires large joint excursions, substantial muscle–tendon length changes, and different transfers between muscle work, elastic energy, and body potential energy. It is therefore important to clarify whether the authors considered that the same assistive strategy may interact differently with musculotendon function in STST versus gait. Even if no direct or model-based musculotendon analysis was performed, it would be highly valuable to acknowledge this task-specific difference in the Discussion
- COF trajectory provides useful information on the distribution and progression of the resultant foot-ground interaction, but by itself it does not capture whole-body dynamic stability, since it does not account for centre of mass motion, trunk compensation, or the CoF/CoM relationship during the transition from seat-off to full extension. I therefore recommend the authors to provide additional justification explaining why COF trajectory alone is considered adequate in this context.

(Remarks on code availability)

Reviewer #2

(Remarks to the Author)
The manuscript presents a soft tendon-driven hip exosuit designed to assist older adults during sit-to-stand transitions and walking activities. The authors conducted a feasibility study with ten community-dwelling older adults to evaluate the device's potential to improve functional mobility. According to the reported results, the exosuit increased performance in sit-to-stand test, reduced the duration of sit-to-stand cycles, and decreased the metabolic cost of walking, while maintaining natural lower-limb kinematics, stability, and a high sense of user agency regarding the ExoOff condition. These findings suggest that textile-based soft robotic systems may represent a promising approach to support mobility and independence in ageing populations. However, although the study presents encouraging results regarding the proposed exosuit, experimental limitations limit a more comprehensive assessment of the true potential of the technology.
My main concern relates to the absence of performance metrics collected without the exosuit. It is unclear how the authors can claim that the exosuit improves efficiency or assists movement if no comparison is provided between conditions without the device and with the device. Comparing the exo-off and exo-on conditions is potentially biased. When a device is added to the body, especially when it is not providing assistance, it is expected that participants may experience greater difficulty performing movements compared to when the system is actively assisting. Therefore, the reported results only support the

conclusion that the powered assistance may partially compensate for the additional load imposed by the device itself. However, they are not sufficient to conclude that the exosuit improves the participants' movement capability relative to a baseline condition without the device.
Throughout the manuscript, the authors repeatedly state that "the exosuit assistance significantly reduced the metabolic cost of transport in older adults," that "assistance provided by the exosuit significantly enhanced sit-and-stand transition performance," and that "the exosuit improves sit-and-stand efficiency while maintaining postural stability in older participants." However, these claims appear to be unsupported since the study does not include measurements of these metrics when participants perform the tasks without wearing the exosuit. Furthermore, since no data collection was performed without the exosuit, it is unclear how the authors can claim that natural lower-limb biomechanics were preserved.
Although the exosuit is described as soft and lightweight system (3.0 kg), this mass may represent nearly a 5% increase in body mass for some participants, which increases the metabolic cost of transport, particularly in older adults. Work rate increases in an approximately linear fashion with added load, rising by about 1.40 W for every additional kilogram carried (see: J Exp Biol . 2014 Feb 15;217(Pt 4):605-13. doi: 10.1242/jeb.091587. Epub 2013 Nov 6.). In addition, even when the device is unpowered, participants still need to exert force to move the actuators due to internal friction and the apparent inertia of the actuator, which may further increase the difficulty of movement in the passive condition.

Minor comments
In Fig. 2E, what is the scale of the x-axis? Does it represent the percentage of the gait cycle (% of gait cycle)?
I found the results presented in Fig. 5 difficult to interpret. The authors may consider presenting the graphs in a more intuitive way to facilitate the reader's understanding.

(Remarks on code availability)

Version 1:

Reviewer comments:

Reviewer #1

(Remarks to the Author)
All my recommendations have been fulfilled.

(Remarks on code availability)

Reviewer #2

(Remarks to the Author)
In this revised version of the manuscript, the authors included a new set of experiments involving participants without wearing the exosuit, and the obtained results are consistent with the expected outcomes and provide additional support for the authors' conclusions. In addition, the manuscript has been appropriately revised, and the authors have satisfactorily addressed all of my comments and concerns. I would like to congratulate the authors on their work and on the efforts made to improve the manuscript.

(Remarks on code availability)

Open Access This Peer Review File is licensed under a Creative Commons Attribution 4.0 International License, which permits use, sharing, adaptation, distribution and reproduction in any medium or format, as long as you give appropriate credit to the original author(s) and the source, provide a link to the Creative Commons license, and indicate if changes were made.
In cases where reviewers are anonymous, credit should be given to 'Anonymous Referee' and the source.
The images or other third party material in this Peer Review File are included in the article's Creative Commons license, unless indicated otherwise in a credit line to the material. If material is not included in the article's Creative Commons license and your intended use is not permitted by statutory regulation or exceeds the permitted use, you will need to obtain permission directly from the copyright holder.
To view a copy of this license, visit https://creativecommons.org/licenses/by/4.0/

2 Response to Reviewer 1

2.1 Comment

In this paper the authors set forth present a soft tendon-driven hip exosuit designed to support sit-to-stand transition (STST) and walking for older adults with reduced lower-limb functional performance. Although this is a well written paper and on an interesting area that is within the scope of the journal further revisions are required ahead of publication.

2.1.1 Reply

We extend our gratitude to the Reviewer for the invaluable feedback and acknowledgment of our work. The insights shared regarding the manuscript are deeply appreciated, and we have worked to address all concerns in the revised version.

The Reviewer can find detailed point-by-point responses below. Modifications were clearly marked within the manuscript and also reported here.

2.2 Comment

The manuscript reports improved STST, but it remains unclear how the exosuit produces this benefit from a biomechanical perspective. STST begins from a highly flexed posture in which the lower limb operates with poor leverage, large joint ranges of motion, and likely reduced effective mechanical advantage (EMA), especially in the early phase and around seat-off. I therefore encourage the authors to clarify whether they assessed phase-specific joint mechanics during STS and a discussion of posture-dependent leverage and EMA would substantially strengthen the mechanistic interpretation.

2.2.1 Reply

We thank the Reviewer for pointing out this insightful comment. Admittedly, assisting sit-to-stand transitions with a soft tendon-driven device is challenging because the biomechanical conditions are less mechanically favorable, particularly during the early flexed posture and around seat-off. However, our system was not designed to fully lift the user from the seated posture or to replace the user's voluntary effort. Instead, the exosuit provides user-cooperative, partial hip-extension assistance that is activated after detecting the user's movement intention. As described in the Controller section in the main manuscript, the system identifies the Sitting state based on the composite joint angle and angular velocity, and assistance is triggered once the composite angle exceeds the

predefined threshold of AngSum $>$ 0.8 rad (sum of the left and right hip angle), corresponding to approximately 25% of the sit-to-stand transition progression. Consequently, the earliest phase of the movement, highlighted by the Reviewer, remains primarily driven by the user's own neuromuscular effort, while the exosuit provides partial hip-extension support during the subsequent extension-dominant phase. The improved sit-and-stand performance can be interpreted as a functional benefit of partial assistance that helps reduce the effort required to complete the transition, rather than as evidence that the exosuit fully compensates for the biomechanical disadvantage of the early flexed posture.

We fully agree with the Reviewer that further clarification and discussion of phase-specific joint mechanics, posture-dependent leverage, and effective mechanical advantage would be highly valuable for better understanding how the wearable assistive device interacts with the biomechanics of sit-and-stand movement. In response to the Reviewer's suggestion, we have clarified the assistance rationale in the Methods-Controller section and highlighted this point as a limitation in the Discussion in the main manuscript, noting that future analyses of posture-dependent leverage and effective mechanical advantage would help strengthen the mechanistic interpretation of the observed sit-and-stand benefits provided by the exosuit. The text is highlighted in green in the manuscript and also reported here:

Methods section (lines 495-506): "The FSM comprises two states: State 1, which includes the Reset and Walking sub-states, and State 2, corresponding to the Sitting state. The system initializes in the Reset sub-state of State 1, representing an upright posture at the onset of locomotion, and remains in the Walking sub-state of State 1 as long as the composite joint angle satisfies AngSum$\leq$0.8 rad. The system switches from Walking to Sitting when AngSum$>$0.8 rad and the composite angular velocity VelSum$\geq$0 rad s^{-1}, indicating a continued increase in joint flexion characteristic of the descent into a seated posture. For sit-and-stand movement, this composite angle threshold corresponds to approximately 25% of the sit-to-stand transition progression, or about 23° for each thigh, and partial hip-extension assistance is provided during the remaining phase of the transition. The transition back to Walking is triggered when AngSum$\leq$2 rad and VelSum$\leq$0 rad s^{-1}, corresponding to the restoration of an upright posture after completion of the sit-to-stand transition."

Discussion section (lines 364-382): "Another point to note is that the sit-to-stand transition begins from a highly flexed posture, where lower-limb leverage is less favorable and effective mechanical advantage may be reduced, particularly around seat-off. Although exosuit assistance produced measurable functional benefits during sit-to-stand transitions, assisting this task with a soft tendon-driven device is inherently challenging from a biomechanical perspective. However, the exosuit was not designed to fully generate the movement or replace the user's voluntary effort. Instead, assistance was delivered after recognition of the user's movement intention and was intended to provide partial hip-extension support during the transition. Thus, movement initiation and the early preparatory phase remained primarily user-driven, while the exosuit contributed assistance during the subsequent extension-dominant phase. The observed improvement in 1-minute sit-to-stand repetitions should therefore be interpreted as a functional benefit of cooperative partial assistance, rather than full mechanical replacement of lower-limb effort. Future studies that combine inverse dynamics with biomechanical modeling could help further clarify how joint mechanics, leverage, and effective mechanical advantage change across different movement phases, thereby strengthening the mechanistic interpretation of the observed improvements and informing the design of wearable assistive systems."

2.3 Comment

Authors present benefits for both STST and walking, but these two tasks differ fundamentally in mechanical and neuromuscular demands. Walking often benefits from near-isometric muscle action and elastic tendon behavior during stance, whereas STST requires large joint excursions, substantial muscle–tendon length changes, and different transfers between muscle work, elastic energy, and body potential energy. It is therefore important to clarify whether the authors considered that the same assistive strategy may interact differently with musculotendon function in STST versus gait. Even if no direct or model-based musculotendon analysis was performed, it would be highly valuable to acknowledge this task-specific difference in the Discussion.

2.3.1 Reply

We agree with the Reviewer that sit-to-stand transitions and walking differ fundamentally in their mechanical and neuromuscular demands, and that the same exosuit assistance strategy may affect the musculoskeletal system differently in these two activities.

In the present study, we adopted a task-oriented control framework, such that sit-to-stand and walking movements were assisted using different control strategies rather than a single undifferentiated assistive mode across different activities. As shown in the figure below (Fig. S2), the exosuit controller consists of a locomotion-mode detection module and a trajectory-generation module. The locomotion mode is identified based on bilateral hip joint angle and velocity features. After gait phase estimation, the trajectory generator produces task-specific motor reference trajectories according to the identified mode. Specifically, for walking, the predicted gait phase Y is scaled by a gain factor $k \in [1, 5]$ to generate the target motor trajectory, expressed as $R_{traj} = k * Y$. The gain factor k can be adjusted according to individual user needs to provide personalized assistance. For sit-to-stand transitions, a different control law is used: the hip angular velocity v_{rt} is used as the control variable to define the reference trajectory, expressed as $R_{traj} = 2 * v_{rt}$. All threshold values were empirically determined by analyzing the correlation and variability of kinematic features across different locomotion modes, sensor noise, and hardware settings, and were guided by prior domain knowledge to optimize control performance and generalization. The reference trajectory is passed through a second-order low-pass filter with a cutoff frequency of 4.8 Hz to ensure the continuity and stability of the generated motion profiles. Fig. S2 also shows the motor reference trajectories and motor torque profiles across different locomotion modes for a representative participant. Compared with walking, the magnitude of assistance delivered during sit-to-stand transitions is higher.

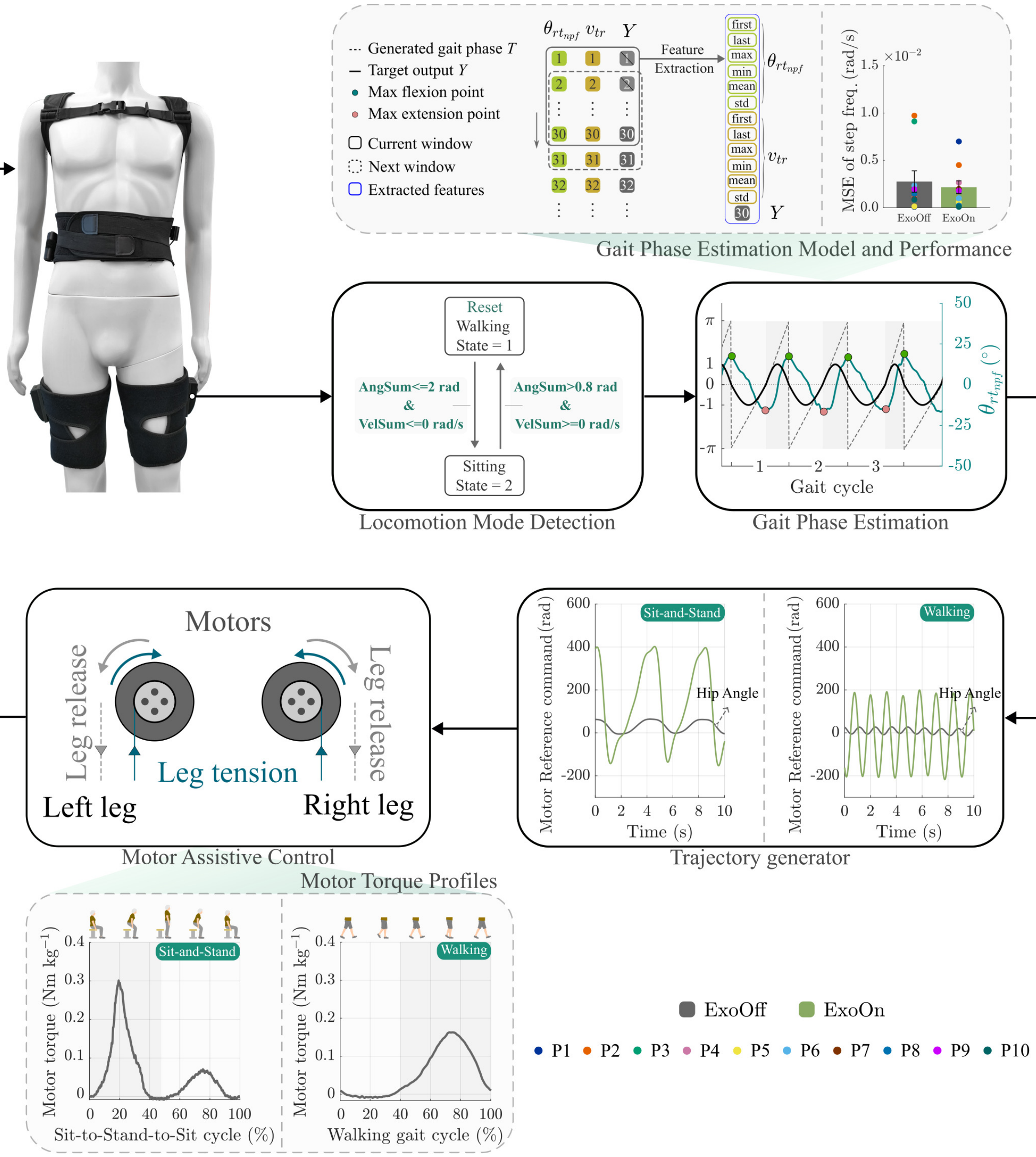

Fig. S2: Schematic diagram and detailed illustration of the controller's working principle.

To this end, we have updated Fig. S2 in the Supplementary Materials to more clearly illustrate the controller structure and task-specific assistance strategies. Furthermore, we agree with the Reviewer that the interaction between the exosuit and the musculoskeletal system may differ between sit-and-stand and walking movements because these two activities involve distinct mechanical and neuromuscular demands. Following the Reviewer's suggestion, we have revised the Discussion section to further highlight this point. The detailed text is as follows.

Discussion section (lines 382-390):"Moreover, it should be noted that sit-and-stand and walking movements involve different mechanical and neuromuscular demands. Although both activities benefited from exosuit assistance under a task-oriented control framework, task-specific demands may lead to distinct interactions between the exosuit and the musculoskeletal system. Future studies incorporating electromyography or musculoskeletal modeling would help clarify how exosuit

assistance influences musculotendon function differently during sit-and-stand and walking tasks."

2.4 Comment

COF trajectory provides useful information on the distribution and progression of the resultant foot-ground interaction, but by itself it does not capture whole-body dynamic stability, since it does not account for centre of mass motion, trunk compensation, or the CoF/CoM relationship during the transition from seat-off to full extension. I therefore recommend the authors to provide additional justification explaining why COF trajectory alone is considered adequate in this context.

2.4.1 Reply

We are grateful to the Reviewer for highlighting that the centre-of-force trajectory alone cannot fully characterize whole-body dynamic stability during the sit-to-stand transitions. In the revised manuscript, we have included trunk kinematic information (Fig. 3 in the revised manuscript, also shown below) and interpreted trunk data together with hip angle and angular velocity as complementary evidence for assessing whether exosuit assistance altered movement patterns and stability-related behaviour during sit-and-stand movement. Results show that, across both the sit-and-stand and walking tasks, hip joint and trunk kinematics were preserved with exosuit assistance. Angle trajectories, ranges of motion, and peak movement velocities showed only small observable changes and did not differ significantly between the *ExoOff* and *ExoOn* conditions. We further clarify in the Discussion section that the centre-of-force trajectory was used as a stability-related indicator of foot-ground interaction during repeated sit-and-stand cycles, rather than as a standalone or comprehensive measure of whole-body dynamic stability.

Accordingly, we have reorganized and represented the kinematic and stability-related results in the Results section and clarified the corresponding discussion in the Discussion section, as follows:

Results section (lines 153-178): "Lower-limb kinematics and stability-related measures during sit-and-stand and walking were analyzed and compared between assisted and unassisted conditions to assess potential alterations in natural movement associated with exosuit assistance. Across both the sit-and-stand and walking tasks, hip joint and trunk kinematics were largely preserved with exosuit assistance. Hip and trunk angle trajectories, ranges of motion, and peak movement velocities showed only small observable changes and did not differ significantly between the *ExoOff* and *ExoOn* conditions (Fig. 3). During sit-and-stand task (Fig. 3A and B), hip angle range of motion increased from $68.47 \pm 1.66\ ^\circ$ in the *ExoOff* condition to $67.86 \pm 1.45\ ^\circ$ in the *ExoOn* condition, and hip peak velocity increased from $60.90 \pm 7.71\ ^\circ s^{-1}$ to $70.11 \pm 7.90\ ^\circ s^{-1}$ for the maximum peak and from $-71.25 \pm 8.54\ ^\circ s^{-1}$ to $-82.06 \pm 7.29\ ^\circ s^{-1}$ for the minimum peak; Trunk range of motion decreased from $40.75 \pm 5.14^\circ$ (*ExoOff*) to $35.45 \pm 3.75^\circ$ (*ExoOn*), while trunk peak velocity changed from $77.31 \pm 5.79^\circ s^{-1}$ to $70.57 \pm 5.43^\circ s^{-1}$ for the maximum peak and from $-68.13 \pm 5.90^\circ s^{-1}$ to $-63.38 \pm 5.66^\circ s^{-1}$ for the minimum peak. During walking (Fig. 3C), Hip angle range of motion changed from $39.75 \pm 1.27\ ^\circ$ to $38.35 \pm 1.30\ ^\circ$, whereas hip peak velocity changed from

164.36 ± 7.52 °s^{-1} to 150.28 ± 5.55 °s^{-1} for the positive peak and from −93.36 ± 5.28 °s^{-1} to −89.37 ± 6.10 °s^{-1} for the negative peak, in the *ExoOff* and *ExoOn* conditions, respectively. The preserved hip and trunk kinematics under exosuit assistance also provided complementary information relevant to the interpretation of movement stability and postural-control-related behavior.

To further characterize stability-related behavior during the sit-and-stand task, the 95% confidence ellipse of the centre-of-force (COF) trajectory recorded from the ground reaction force platform was analyzed. The semi-major and ...”

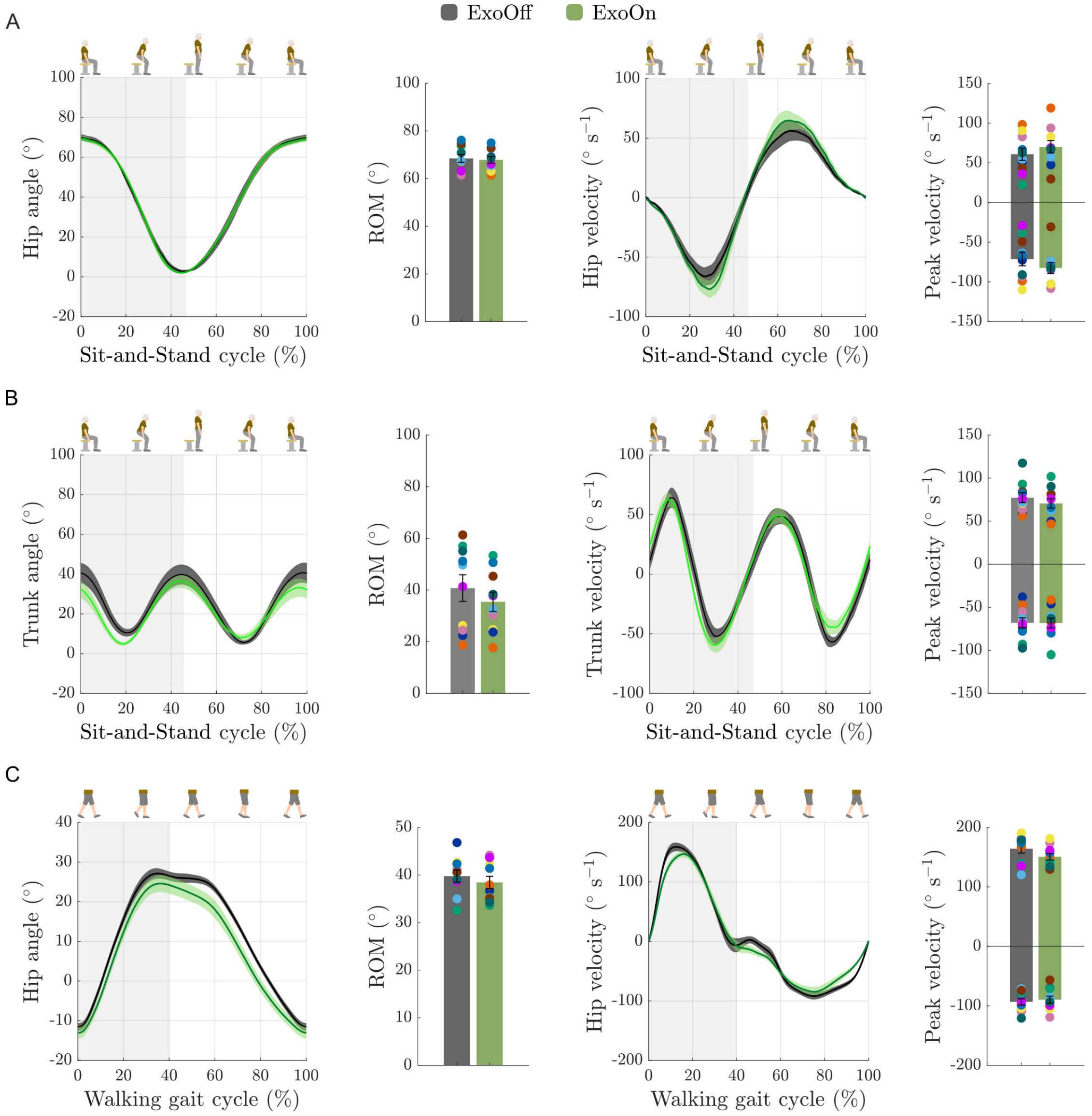

Fig. 3: Kinematic evaluation during sit-and-stand and walking movements in older adults. Angle and velocity profiles during sit-and-stand and walking tasks: **A** Hip and **B** trunk profiles during sit-and-stand; **C** hip profile during walking. Range of motion (ROM) was calculated as the difference between the maximum and minimum angles within each segmented sit-and-stand or walking cycle across steps and participants. Velocity profiles were quantified by peak positive and peak negative velocity values. Bar plots show the mean ± s.e.m. for the *ExoOff* (grey) and *ExoOn* (green) conditions, and overlaid dots represent data from individual participants.

Discussion section (lines 302-312): "Lower-limb and trunk kinematic analysis further supports the result that exosuit assistance did not substantially alter the fundamental movement pattern of the sit-and-stand movement or markedly compromise stability. However, it should be noted that the 95% confidence ellipse area of the centre of force (COF) trajectory, used here as a stability-related measure during repeated sit-and-stand cycles, primarily reflects foot-ground interaction characteristics. A more comprehensive assessment of whole-body dynamic stability would require additional biomechanical measures, including centre-of-mass displacement and velocity and continuous changes in the relationship between the centre of mass and the base of support during the sit-and-stand movement."

3 Response to Reviewer 2

3.1 Comment

> The manuscript presents a soft tendon-driven hip exosuit designed to assist older adults during sit-to-stand transitions and walking activities. The authors conducted a feasibility study with ten community-dwelling older adults to evaluate the device's potential to improve functional mobility. According to the reported results, the exosuit increased performance in sit-to-stand test, reduced the duration of sit-and-stand cycles, and decreased the metabolic cost of walking, while maintaining natural lower-limb kinematics, stability, and a high sense of user agency regarding the *ExoOff* condition. These findings suggest that textile-based soft robotic systems may represent a promising approach to support mobility and independence in ageing populations.
> However, although the study presents encouraging results regarding the proposed exosuit, experimental limitations limit a more comprehensive assessment of the true potential of the technology. My main concern relates to the absence of performance metrics collected without the exosuit. It is unclear how the authors can claim that the exosuit improves efficiency or assists movement if no comparison is provided between conditions without the device and with the device. Comparing the exo-off and exo-on conditions is potentially biased. When a device is added to the body, especially when it is not providing assistance, it is expected that participants may experience greater difficulty performing movements compared to when the system is actively assisting. Therefore, the reported results only support the conclusion that the powered assistance may partially compensate for the additional load imposed by the device itself. However, they are not sufficient to conclude that the exosuit improves the participants' movement capability relative to a baseline condition without the device.

3.1.1 Reply

We thank the Reviewer for the overall evaluation of our work and for pointing out that the absence of a No-Exosuit (*NoExo*) baseline limited a more comprehensive assessment of the exosuit's assistive effect.

To address this concern, we recruited 7 of the 10 original participants who were available for a further experimental session. These 7 older participants repeated the same protocol described in the main manuscript without wearing the exosuit (*NoExo*), while retaining only the recording unit with IMU sensors and the metabolic analyzer to enable kinematic and metabolic recordings and comparisons. Comparisons of the measured repetitions, metabolic cost, kinematic, and biomechanical outcomes were then performed between the *NoExo* and *ExoOn* conditions. As presented below, results from the *NoExo* baseline and *ExoOn* condition showed trends broadly consistent with the *ExoOff* and *ExoOn* findings in the main manuscript, suggesting that the relatively low weight of the device (2.86 kg), together with its placement near the body centre of mass rather than distally on the lower limbs, did not substantially affect the measured outcomes. In consideration of this reduced sample size, in the first implementation of our study, we decided to present these results

in the Supplementary Materials, whereas the main manuscript retains the original two-condition comparison: *ExoOff* and *ExoOn*, in line with the experimental framework commonly adopted in the existing literature on wearable robotic assistance [1, 2, 3, 4].

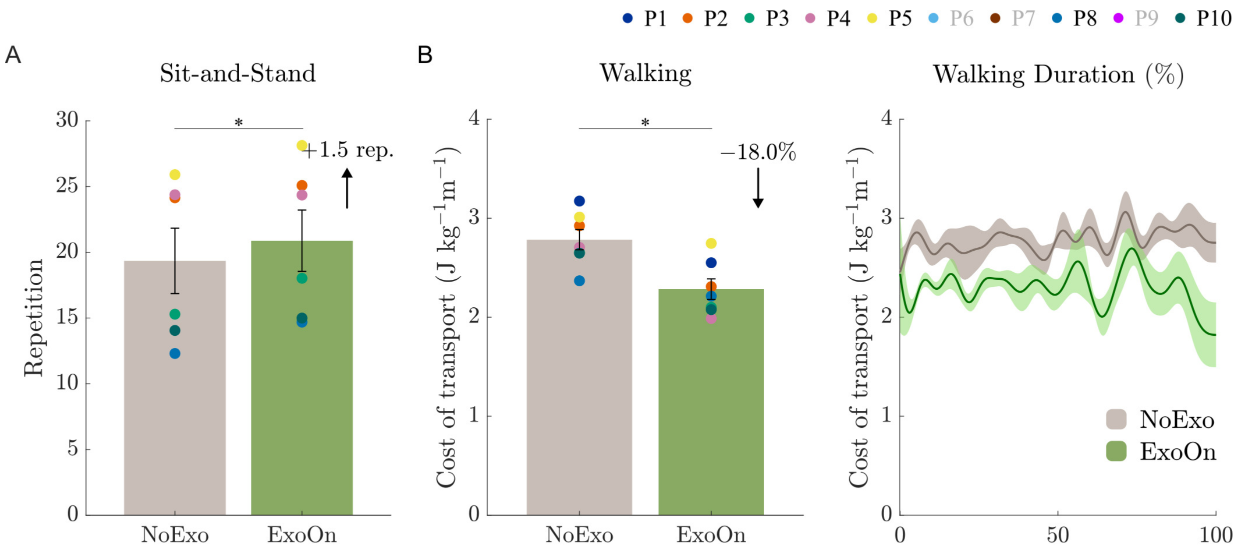

Fig. S7: Performance evaluation of the exosuit in older adults under *NoExo* and *ExoOn* conditions. A Total repetitions in 1-minute sit-to-stand test. **B** Steady Walking metabolic cost of transport measured during the 6-minute walking test. Bars indicate mean ± s.e.m., with light brown and green bars denoting the *NoExo* and *ExoOn* conditions, respectively. Dots represent individual participants, with bold black labels indicating the 7 participants included in this supplementary analysis and grey-shaded labels indicating originally enrolled participants who were unavailable and therefore not included. * denotes a statistically significant difference ($p < 0.05$, paired t-tests).

The results indicate that compared with *NoExo*, exosuit assistance (*ExoOn*) significantly increased 1-minute sit-to-stand performance by an average of 1.5 repetitions across all the participants (Fig. S7A in the Supplementary Materials; $p = 0.017$), compared with the average increase of 1.8 repetitions observed relative to the *ExoOff* condition (Fig. 2B in the main manuscript, $p = 0.006$). During walking, exosuit assistance significantly reduced the metabolic cost of transport by 18.0% (Fig. S7B; $p < 0.001$), compared with the 13.6% reduction observed relative to the *ExoOff* condition in the main manuscript (Fig. 2D; $p = 0.030$). These findings suggest that exosuit assistance compensated for the burden associated with wearing the exosuit in the *ExoOff* condition and provided a measurable net energetic benefit during sustained walking. In addition, exosuit assistance was associated with significant changes only in walking hip range of motion and negative peak angular velocity. However, the overall lower-limb movement pattern remained largely preserved, and force-related stability metrics were maintained (Fig. S8, S9). Together with the original *ExoOff* and *ExoOn* results, these findings further support the conclusion that the soft exosuit enhanced sit-and-stand and walking performance in older adults without substantially altering movement kinematics or compromising stability.

Accordingly, the Supplementary Materials and the corresponding statements in the Discussion section of the main manuscript have been updated to incorporate these additional findings and strengthen the conclusions.

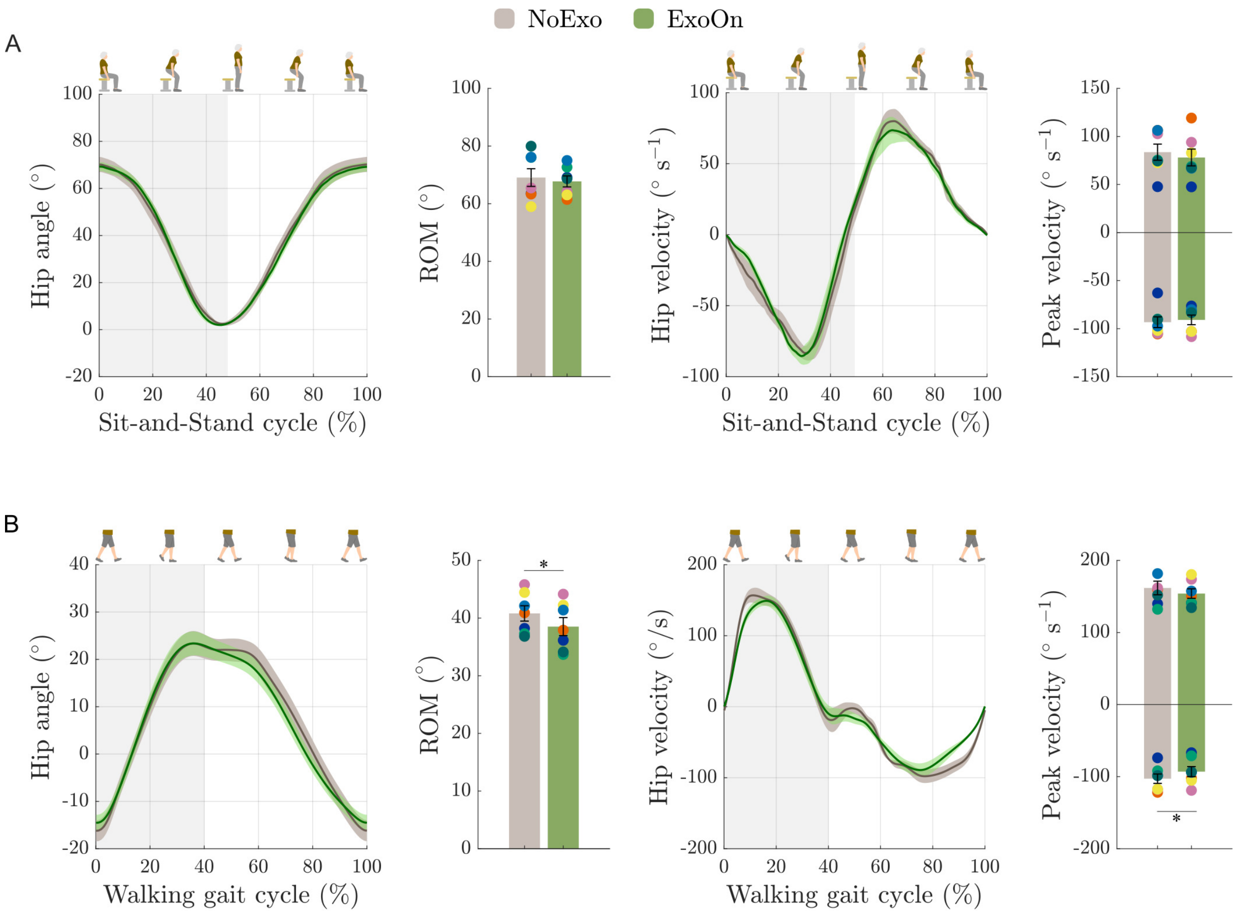

Fig. S8: Kinematic evaluation in older adults under *NoExo* and *ExoOn* conditions. Hip angle and velocity profile during **A** sit-and-stand and **B** walking movements. Range of motion (ROM) was calculated as the difference between the maximum and minimum hip angles within each segmented sit-and-stand or walking cycle across steps and participants. Velocity profiles were quantified by peak positive and peak negative velocity values. Bar plots show the mean ± s.e.m. for the *NoExo* (light brown) and *ExoOn* (green) conditions.

Discussion section (lines 340-364):"A limitation of this study is that the primary experimental design compared only two wearing conditions: *ExoOff* (device worn with assistance disabled) and *ExoOn* (device worn with assistance enabled), without including a no-exosuit condition in the full participant cohort. To further contextualize the effect of active assistance relative to natural, unassisted movement, we also conducted a supplementary experimental session with a subgroup of 7 of the 10 originally enrolled participants, in which the same tasks were completed without wearing the exosuit (*NoExo*). The supplementary findings with *NoExo* and *ExoOn* were broadly consistent with the comparison between the *ExoOff* and *ExoOn* conditions. Specifically, compared with *NoExo*, the *ExoOn* condition significantly increased 1-minute sit-to-stand performance by an average of 1.5 repetitions ($p = 0.017$) and significantly reduced the metabolic cost of transport during the 6-minute walking test by 18.0% ($p < 0.001$) across all the participants, which is comparable to the corresponding improvements of 1.8 repetitions and 13.6% observed under *ExoOff* and *ExoOn* conditions. Given that the exosuit is a soft, lightweight textile-based system (2.86 kg), with its mass concentrated near the user's centre of mass rather than distally on the lower limbs, passive interference from device wearing is expected to be limited. This interpretation is consistent

with our previous observations and related work [5, 3]. Together with the comparable outcomes observed in the *NoExo* analysis, this supports the use of the *ExoOff* condition as a plausible reference baseline for assessing the effect of active assistance. Notably, similar experimental paradigms have been widely adopted in related studies [6, 1, 4]. The detailed results related to *NoExo* are presented in Supplementary Figs. S7-S9."

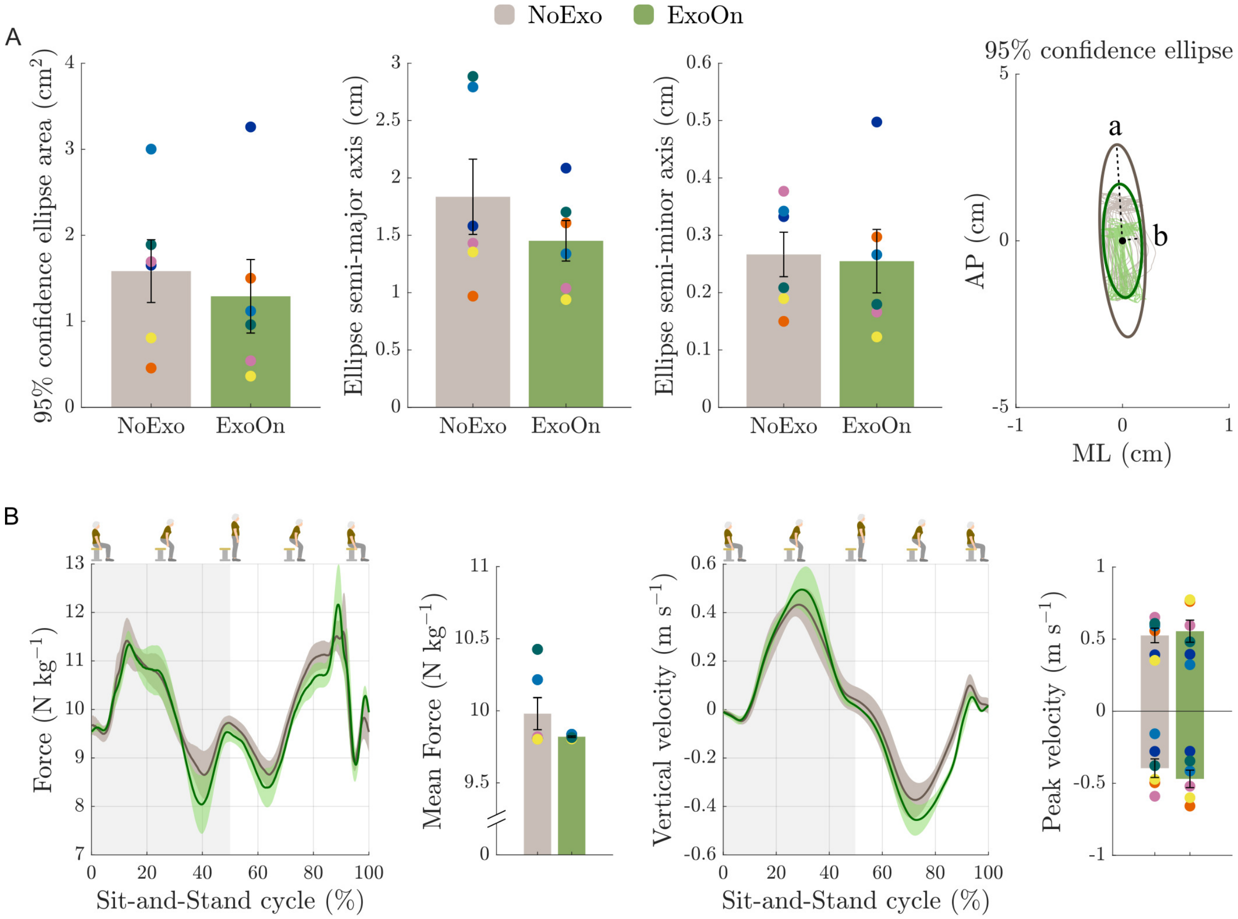

Fig. S9: Biomechanical analysis and stability assessment of sit-and-stand movement in older adults under *NoExo* and *ExoOn* conditions. A 95% confidence ellipse fitted to centre of force (COF) data points across participants during sit-to-stand test. The lengths of the semi-major axis (a) and semi-minor axis (b) also served as metrics for quantifying postural sway of the plantar pressure in the anteroposterior (AP) and mediolateral (ML) directions, respectively. Data from a representative participant are shown for illustration, with AP and ML coordinates zero-centered. **B** Trajectory and mean vertical ground reaction force and velocity across older participants. Bar plots show mean ± s.e.m.; dots indicate individual participants.

Supplementary Materials (lines 169-179): "**Participants**: To provide a baseline without wearing the exosuit (*NoExo*), a further experimental session was conducted in a subgroup of 7 of the 10 originally enrolled participants (3 females and 4 males; age 69–85 years, mean 79.0 ± 6.0 years; height 171.7 ± 5.0 cm; weight 66.3 ± 3.4 kg, mean ± SD).

In this supplementary experiment, participants repeated the same protocol (1-minute sit-to-stand test and 6-minute walking test) described in the main manuscript without wearing the exosuit,

while retaining only the recording unit with IMU sensors and the metabolic analyzer to enable kinematic and metabolic recordings and comparisons. Accordingly, comparisons among *NoExo* and *ExoOn* conditions reported in this section were performed within this subgroup."

Supplementary Materials (lines 225-244): "**Conclusion:** Results from the *NoExo* and *ExoOn* conditions showed trends broadly consistent with those observed in the *ExoOff* and *ExoOn* analysis reported in the main manuscript. Compared with *NoExo*, exosuit assistance (*ExoOn*) significantly increased 1-minute sit-to-stand performance by an average of 1.5 repetitions across all the participants (Fig. S7A; $p = 0.017$), compared with the average increase of 1.8 repetitions observed relative to the *ExoOff* condition (Fig. 2B in the main manuscript, $p = 0.006$). During walking, exosuit assistance significantly reduced the metabolic cost of transport by 18.0% (Fig. S7B; $p < 0.001$), compared with the 13.6% reduction observed relative to the *ExoOff* condition in the main manuscript (Fig. 2D; $p = 0.030$). These findings suggest that exosuit assistance compensated for the burden associated with wearing the exosuit in the *ExoOff* condition and provided a measurable net energetic benefit during sustained walking. In addition, exosuit assistance was associated with significant changes only in walking hip range of motion and negative peak angular velocity. However, the overall lower-limb movement pattern remained largely preserved, and force-related stability metrics were maintained (Fig. S8, S9). These findings further support the conclusion that the soft exosuit enhanced sit-and-stand and walking performance in older adults without substantially altering movement kinematics or compromising stability."

3.2 Comment

Throughout the manuscript, the authors repeatedly state that "the exosuit assistance significantly reduced the metabolic cost of transport in older adults," that "assistance provided by the exosuit significantly enhanced sit-and-stand transition performance," and that "the exosuit improves sit-and-stand efficiency while maintaining postural stability in older participants." However, these claims appear to be unsupported since the study does not include measurements of these metrics when participants perform the tasks without wearing the exosuit. Furthermore, since no data collection was performed without the exosuit, it is unclear how the authors can claim that natural lower-limb biomechanics were preserved.

3.2.1 Reply

We thank the reviewer for the suggestions. We agree that the absence of a *NoExo* baseline in the original study limited the interpretation of the effects of exosuit assistance reported in the manuscript. As detailed in our response to Comment 3.1, we have now included a comparison between the *NoExo* and *ExoOn* conditions in a subgroup of 7 originally enrolled older participants. The supplementary analysis offers a reference for interpreting the effects of active assistance (*ExoOn*) in comparison to natural, unassisted movement (*NoExo*), with respect to the number of repetitions, metabolic cost of walking, kinematics, and stability-related outcomes. The supplementary results are broadly consistent with the main comparison between *ExoOff* and *ExoOn*, supporting the

interpretation that the exosuit improved sit-and-stand performance and reduced walking metabolic cost while preserving the users' original movement patterns and stability.

The detailed results of the comparison between the *NoExo* and *ExoOn* conditions are shown below and are also reported in the Supplementary Materials (Supplementary Figs. S7–S9).

Supplementary Materials (lines 180-224): Effect of Exosuit Assistance Compared with No-Exosuit Condition

"**Sit-and-stand efficiency was found to be significantly improved in older adults.** The comparison between the *NoExo* and *ExoOn* conditions indicated that exosuit assistance enhanced sit-to-stand transition performance. During the 1-minute sit-to-stand test, participants completed 1.5 $\pm$ 0.4 more cycles in the *ExoOn* condition than in the *NoExo* condition (19.4 $\pm$ 2.5 repetitions in *NoExo* and 20.9 $\pm$ 2.3 repetitions in *ExoOn*; $p = 0.017$; Fig. 7A, left). Exosuit assistance also shortened sit-and-stand cycle duration, with a maximum individual reduction of 16.3% (4.87 s in *NoExo* and 4.08 s in *ExoOn*).

Metabolic cost of transport during walking was significantly reduced with exosuit assistance. Exosuit assistance significantly reduced the metabolic cost of transport in older adults, with a mean reduction of 18.0% across participants (from 2.78 $\pm$ 0.10 $Jkg^{-1}m^{-1}$ to 2.28 $\pm$ 0.10 $Jkg^{-1}m^{-1}$; $p < 0.001$) (Fig. 7B). All participants exhibited a lower metabolic cost of transport with exosuit assistance. The largest individual reduction reached 26.5%, decreasing from 2.70 $Jkg^{-1}m^{-1}$ to 1.99 $Jkg^{-1}m^{-1}$.

Lower-limb natural kinematics and stability were largely preserved. Lower-limb kinematics and stability-related measures during sit-and-stand and walking tasks were also analyzed and compared between *NoExo* and *ExoOn* conditions to assess potential alterations in natural movement associated with exosuit assistance. Across both sit-and-stand and walking tasks, hip joint kinematics remained broadly comparable with exosuit assistance. During the sit-and-stand task (Fig. 8A), hip angle range of motion changed from 69.08 $\pm$ 3.07 $^\circ$ in the *NoExo* condition to 67.74 $\pm$ 1.91 $^\circ$ in the *ExoOn* condition. Hip peak velocity changed from 83.66 $\pm$ 8.40 $^\circ s^{-1}$ to 78.11 $\pm$ 7.85 $^\circ s^{-1}$ for the positive peak, and from -93.28 ± 5.60 $^\circ s^{-1}$ to -90.87 ± 5.00 $^\circ s^{-1}$ for the negative peak. During walking (Fig. 8B), hip angle range of motion was significantly different between the *NoExo* and *ExoOn* conditions, changing from 40.81 $\pm$ 1.34 $^\circ$ to 38.52 $\pm$ 1.56 $^\circ$ ($p < 0.001$). Hip peak velocity changed from 161.78 $\pm$ 9.44 $^\circ s^{-1}$ to 154.05 $\pm$ 6.62 $^\circ s^{-1}$ for the positive peak, whereas the negative peak velocity was also significantly different, changing from -102.80 ± 6.52 $^\circ s^{-1}$ to -93.05 ± 7.02 $^\circ s^{-1}$ ($p = 0.021$).

The 95% confidence ellipse of the centre-of-force (COF) trajectory measured from the ground reaction force platform was further analyzed, and no significant differences were observed between the *NoExo* and *ExoOn* conditions. As shown in Fig. 9A, the COF ellipse area was 1.58 $\pm$ 0.37 cm^2 in *NoExo* and 1.29 $\pm$ 0.43 cm^2 in *ExoOn*. The semi-major axis length in the AP direction was 1.84 $\pm$ 0.33 cm and 1.45 $\pm$ 0.18 cm, whereas the minor axis length in the ML direction was 0.27 $\pm$ 0.04 cm and 0.25 $\pm$ 0.06 cm, respectively. In addition, the total ground reaction force and centre-of-mass velocity profiles (Fig. 9B) were similar across the two conditions. Mean ground reaction force was 9.98 $\pm$ 0.11 Nkg^{-1} in *NoExo* and 9.82 $\pm$ 0.005 Nkg^{-1} in *ExoOn*. Peak centre of mass velocity also showed only small differences, with maximum values of 0.53 $\pm$ 0.05 ms^{-1} and 0.55 $\pm$ 0.08 ms^{-1} and minimum values of -0.40 ± 0.06 ms^{-1} and -0.47 ± 0.06 ms^{-1} in the *NoExo* and *ExoOn* conditions, respectively."

3.3 Comment

Although the exosuit is described as soft and lightweight system (3 kg), this mass may represent nearly a 5% increase in body mass for some participants, which increases the metabolic cost of transport, particularly in older adults. Work rate increases in an approximately linear fashion with added load, rising by about 1.40 W for every additional kilogram carried (see: J Exp Biol . 2014 Feb 15;217(Pt 4):605-13. doi: 10.1242/jeb.091587. Epub 2013 Nov 6.). In addition, even when the device is unpowered, participants still need to exert force to move the actuators due to internal friction and the apparent inertia of the actuator, which may further increase the difficulty of movement in the passive condition.

3.3.1 Reply

We are grateful to the Reviewer for highlighting the influence of device structure on metabolic outcomes. We fully agree that mechanical work and metabolic demand increase with added load, particularly in older adults.

As described in our responses to Comments 3.1 and 3.2, we addressed this concern by conducting a supplementary experiment in a subgroup of 7 older participants. In this experiment, participants performed the same tasks without wearing the exosuit, providing a *NoExo* baseline for comparison with the *ExoOn* condition. This new comparison allowed us to distinguish the net benefit provided by active assistance. The results show that, compared with *NoExo*, the *ExoOn* condition significantly increased 1-minute sit-to-stand performance by an average of 1.5 repetitions ($p = 0.017$) and reduced the metabolic cost of transport during walking by 18.0% ($p < 0.001$) across all the participants. These changes were comparable to the 1.8 repetitions increase and 13.6% metabolic reduction observed in the original *ExoOff* and *ExoOn* comparison conditions.

At the same time, it is worth noting that the reduced weight of our device (2.86 kg), coupled with its placement at the centre of mass of the body and not on the legs, would have minimal impact on metabolic outcomes for the whole group of participants. In fact, as evidenced by biomechanical studies investigating the impact of additional weights placed on various parts of the lower limbs and waist [[7]], the addition of extra weight at the waist level has been shown to have no significant effect on the metabolic rate, even under high loads. The Reviewer can refer to the figure alongside from Browning et al., which supports the minimal increase in metabolic rate while walking with added masses at the waist, contrary to the effects for the shank, thigh, and foot.

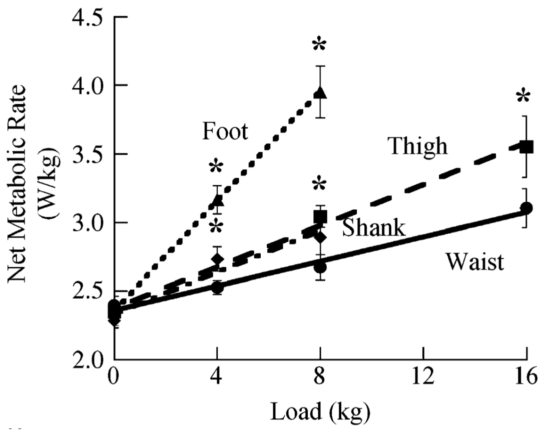

Browning et al. [7]. **Mean net metabolic rate (Wkg^{-1}) vs load mass (m) and location.** The increase in net metabolic rate is greatest with foot loads, similar for thigh and shank loads, and least for waist loads.

Nevertheless, we acknowledge that the *ExoOff* baseline in the original experimental design limited the direct interpretation of the net benefit of active assistance relative to natural, unassisted movement. However, given that the present exosuit is a soft textile-based system rather than a rigid structure, participants were not mechanically constrained by rigid links or frames. Together with the relatively low mass of the device and its placement near the body centre of mass, the effect of device weight on the measured outcomes is expected to be limited, which is also consistent with our previous observations and related work [5, 3]. Importantly, the supplementary *NoExo* and *ExoOn* comparison showed trends broadly consistent with the original *ExoOff* and *ExoOn* findings, with improvements in both sit-and-stand performance and walking metabolic cost. These results support the relevance of the original comparison and indicate that the observed benefits were not merely due to compensation for device-wearing effects, but reflected a meaningful effect of active assistance. To this end, we have revised the Discussion to acknowledge this limitation more clearly while maintaining that the *ExoOff* and *ExoOn* comparison remains informative for evaluating the effect of active assistance.

Discussion section (lines 340-364): "A limitation of this study is that the primary experimental design compared only two wearing conditions: *ExoOff* (device worn with assistance disabled) and *ExoOn* (device worn with assistance enabled), without including a no-exosuit condition in the full participant cohort. To further contextualize the effect of active assistance relative to natural, unassisted movement, we also conducted a supplementary experimental session with a subgroup of 7 of the 10 originally enrolled participants, in which the same tasks were completed without wearing the exosuit (*NoExo*). The supplementary findings with *NoExo* and *ExoOn* were broadly consistent with the comparison between the *ExoOff* and *ExoOn* conditions. Specifically, compared with *NoExo*, the *ExoOn* condition significantly increased 1-minute sit-to-stand performance by an average of 1.5 repetitions ($p = 0.017$) and significantly reduced the metabolic cost of transport during the 6-minute walking test by 18.0% ($p < 0.001$) across all the participants, which is comparable to the corresponding improvements of 1.8 repetitions and 13.6% observed under *ExoOff* and *ExoOn* conditions. Given that the exosuit is a soft, lightweight textile-based system (2.86 kg), with its mass concentrated near the user's centre of mass rather than distally on the lower limbs, passive interference from device wearing is expected to be limited. This interpretation is consistent with our previous observations and related work [5, 3]. Together with the comparable outcomes observed in the *NoExo* analysis, this supports the use of the *ExoOff* condition as a plausible reference baseline for assessing the effect of active assistance. Notably, similar experimental paradigms have been widely adopted in related studies [6, 1, 4]. The detailed results related to *NoExo* are presented in Supplementary Figs. S7-S9."

3.4 Comment

In Fig. 2E, what is the scale of the x-axis? Does it represent the percentage of the gait cycle (% of gait cycle)?

3.4.1 Reply

We appreciate the Reviewer for pointing out that Fig. 2 was unclear. Fig. 2E presents the average walking gait speed across participants during the 6-minute walking test under the *ExoOff* and *ExoOn* conditions. We have revised and clarified Fig. 2 in the revised manuscript and also added figure legends to all the other figures for clarity. The updated Fig. 2 is also shown as follows.

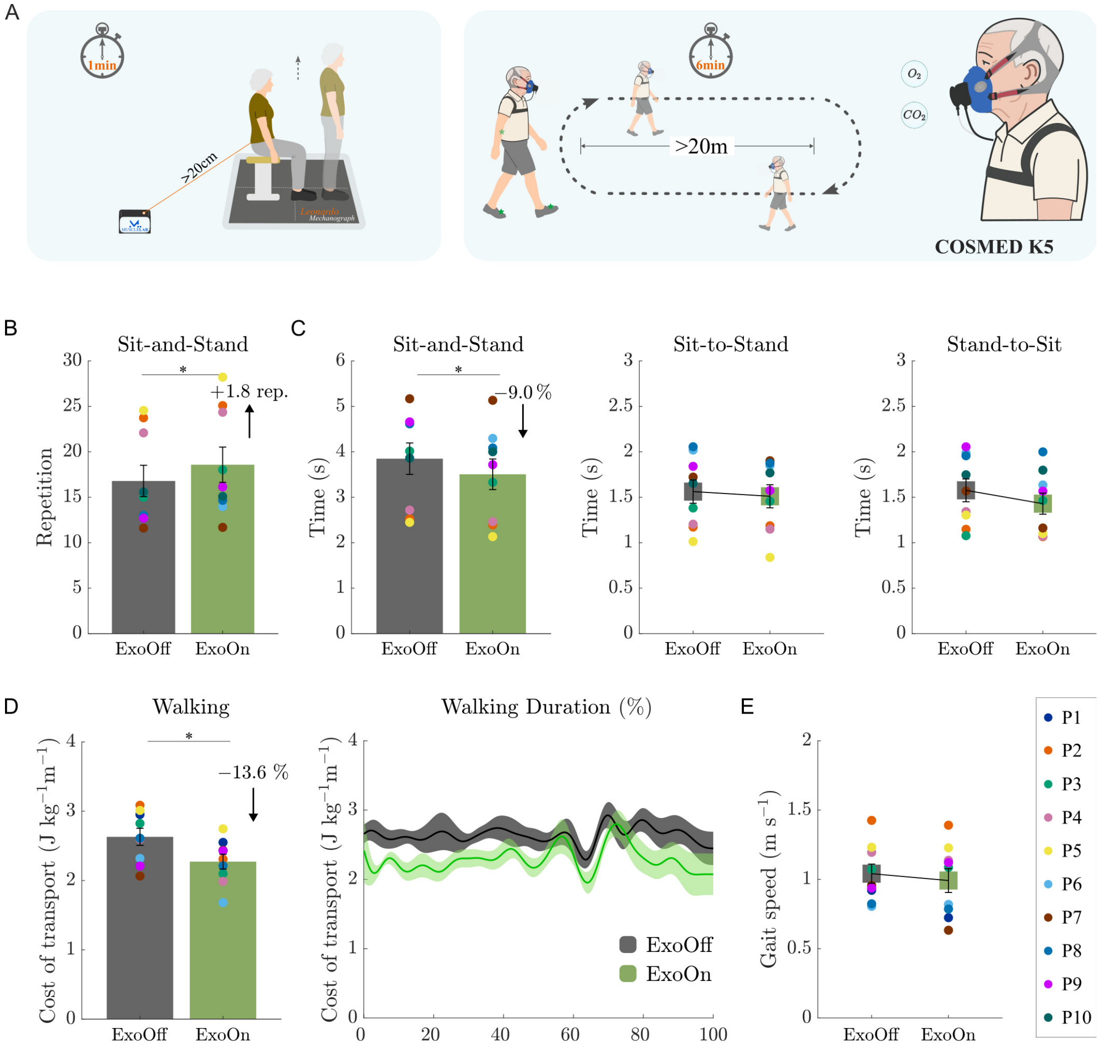

Fig. 2: Experiment and performance evaluation of the exosuit in older adults. A Experiment overview of sit-and-stand and walking. Sit-and-stand performance evaluation in 1-minute sit-to-stand test: **B** the total repetitions and **C** time of each sit-and-stand cycle and transitions across participants. Walking performance evaluation in 6-minute walking test: **D** the walking metabolic cost of transport and **E** average walking gait speed across participants under the *ExoOff* (unassisted) and *ExoOn* (assisted) conditions. The bar plots are presented as mean ± s.e.m., with grey and green bars denoting the *ExoOff* and *ExoOn* conditions, respectively. Colored dots overlaid on the bars represent individual participants. * indicates statistically significant differences ($p < 0.05$, paired t-tests).

3.5 Comment

I found the results presented in Fig.5 difficult to interpret. The authors may consider presenting the graphs in a more intuitive way to facilitate the reader's understanding.

3.5.1 Reply

We thank the Reviewer's suggestion and apologize that the presentation of Fig. 5 was not sufficiently intuitive. We have revised Fig. 5 to present the results in a clearer and more intuitive manner. The corresponding figure caption and the related text in the main manuscript have also been revised to improve readability. The revised figure is also provided below.

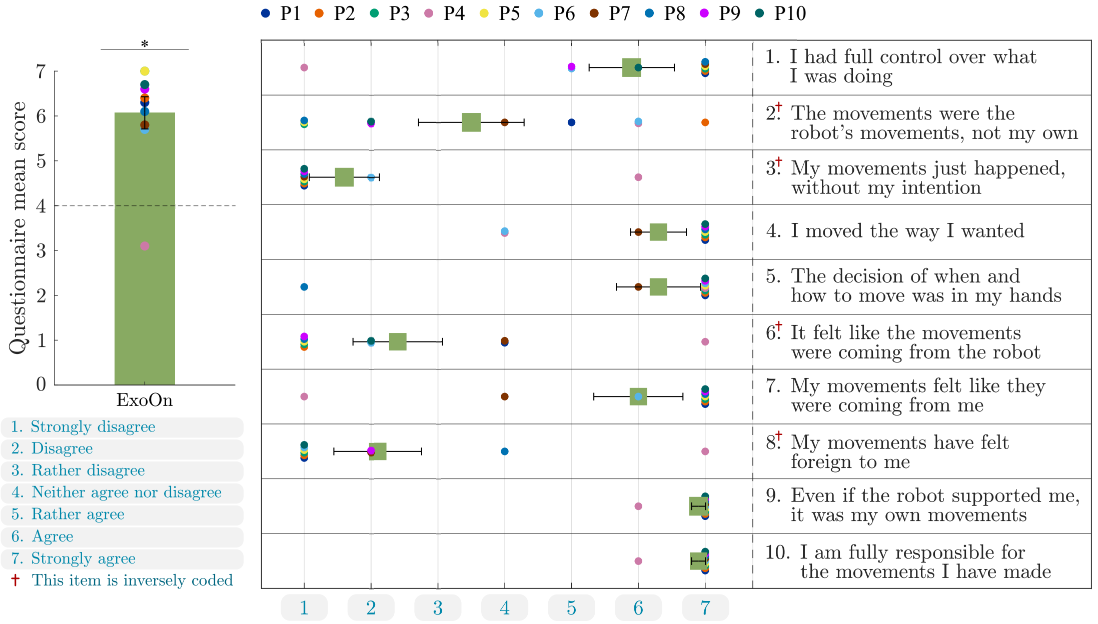

Fig. 5: Sense of agency questionnaire in older adults. Mean score (± s.e.m.; green bar) for each questionnaire item across all participants, along with the distribution of participant responses to each item (1-10) on the 7-point (ranging from "Strongly disagree" to "Strongly agree") sense of agency questionnaire. The y-axis represents each question item, the x-axis represents the Likert response option, while colored circular markers denote individual participants' responses. Items 2, 3, 6, and 8 are inversely coded, whereas items 1, 4, 5, 7, 9, and 10 follow the standard scoring direction.

Results section (lines 205-206): "Older adults reported a high perceived sense of agency during exosuit-assisted sit-and-stand and walking movements, as assessed by a 10-item questionnaire, with a mean score of 6.07 ± 0.38, which was significantly higher than the neutral midpoint of 4 (n = 10, $p < 0.001$; Fig. 5). ...... Fig. 5 also shows the distribution (mean ± s.e.m.) of responses across individual items of the sense of agency questionnaire."

References

[1] F. L. Haufe, E. G. Duroyon, P. Wolf, R. Riener, and M. Xiloyannis, "Outside testing of wearable robots for gait assistance shows a higher metabolic benefit than testing on treadmills," *Scientific reports*, vol. 11, no. 1, p. 14833, 2021.

[2] K. Schmidt, J. E. Duarte, M. Grimmer, A. Sancho-Puchades, H. Wei, C. S. Easthope, and R. Riener, "The myosuit: Bi-articular anti-gravity exosuit that reduces hip extensor activity in sitting transfers," *Frontiers in neurorobotics*, vol. 11, p. 57, 2017.

[3] E. Tricomi, F. Missiroli, M. Xiloyannis, N. Lotti, X. Zhang, M. Stefanakis, M. Theisen, J. Bauer, C. Becker, and L. Masia, "Soft robotic shorts improve outdoor walking efficiency in older adults," 2024.

[4] G. S. Sawicki and D. P. Ferris, "Mechanics and energetics of level walking with powered ankle exoskeletons," *Journal of Experimental Biology*, vol. 211, no. 9, pp. 1402–1413, 2008.

[5] X. Zhang, E. Tricomi, F. Missiroli, N. Lotti, and L. Masia, "Real-time assistive control via imu locomotion mode detection in a soft exosuit: An effective approach to enhance walking metabolic efficiency," *IEEE/ASME Transactions on Mechatronics*, 2023.

[6] M. Xiloyannis, R. Alicea, A.-M. Georgarakis, F. L. Haufe, P. Wolf, L. Masia, and R. Riener, "Soft robotic suits: State of the art, core technologies, and open challenges," *IEEE Transactions on Robotics*, vol. 38, no. 3, pp. 1343–1362, 2021.

[7] R. C. Browning, J. R. Modica, R. Kram, and A. Goswami, "The effects of adding mass to the legs on the energetics and biomechanics of walking," *Medicine & Science in Sports & Exercise*, vol. 39, no. 3, pp. 515–525, 2007.
